# M1C is a druggable target for NSCLC KRAS G12C mutant tumors resistant to KRAS inhibitors

**DOI:** 10.1038/s41388-026-03934-2

**Published:** 2026-08-05

**Authors:** Shinkichi Takamori, Naoki Haratake, Atrayee Bhattacharya, Hiroki Ozawa, Keisuke Shigeta, Mai Onishi, Kentaro Nonaka, Takefumi Komiya, Hiroki Komatsuda, Tomoyoshi Takenaka, Tomoharu Yoshizumi, Chendi Li, Jiehui Deng, Aaron N. Hata, Kwok K. Wong, Mark D. Long, Donald Kufe

**Affiliations:** 1 Department of Medical Oncology Dana-Farber Cancer Institute Harvard Medical School, Boston, MA USA; 2 Division of Hematology and Oncology Penn State College of Medicine, Hershey, PA USA; 3 Department of Surgery and Science Graduate School of Medical Sciences Kyushu University, Fukuoka, Japan; 4 Department of Medicine, Massachusetts General Hospital, Harvard Medical School, Boston, MA USA; 5 Laura and Isaac Perlmutter Cancer Center, New York University Langone Medical Center, New York, NY USA; 6 Department of Biostatistics & Bioinformatics, Roswell Park Comprehensive Cancer Center, Buffalo, NY USA; 7 Present Address: Department of Thoracic and Breast Surgery, Oita University Faculty of Medicine, Oita, Japan; 8 Present Address: University of Texas Health Science Center at Tyler, Tyler, TX USA

**Keywords:** Non-small-cell lung cancer, Targeted therapies

## Abstract

Treatment of NSCLC KRAS G12C mutant tumors with the allele-selective sotorasib and adagrasib inhibitors is invariably associated with acquired resistance. The MUC1-encoded oncogenic M1C protein is necessary for self-renewal of NSCLC KRAS mutant cells. We report that treatment of NSCLC KRAS G12C cells with sotorasib induces M1C expression by a STAT1-dependent pathway. In turn, M1C drives the sotorasib resistant phenotype by NF-κB-mediated induction of the epithelial-mesenchymal transition (EMT) and a mucinous gene program. Targeting M1C→NF-κB signaling (i) suppresses EMT and mucin genes, and (ii) reverses sotorasib resistance. Of translational relevance, treatment with a M1C antibody-drug conjugate (ADC) is effective against two sotorasib-resistant NSCLC KRAS G12C cell lines and two patient-derived tumor xenograft models. Analysis of patients with NSCLC KRAS G12C tumors treated with sotorasib/adagrasib and overexpressing MUC1 associates with decreases in overall survival. These findings identify M1C as a key effector of sotorasib resistance and as a target for treatment of patients with refractory NSCLC KRAS G12C mutant tumors.

## Plain language summary

Resistance of NSCLC KRAS G12C tumors to the FDA-approved sotorasib and adagrasib inhibitors is a major challenge for patient treatment. The basis for this resistance and how to circumvent it have remained unclear. The aim of this study was to determine if the oncogenic M1C protein, which drives NSCLC progression, is responsible for resistance to these agents. Studies of NSCLC cells found that M1C is essential for the sotorasib/adagrasib-resistant phenotype. M1C induces the epithelial mesenchymal transition (EMT) as a mechanism that confers resistance. The significance of these results is supported by the demonstration that targeting M1C reverses sotorasib/adagrasib resistance. These findings identify M1C as a target for the treatment of patients with refractory NSCLC KRAS G12C tumors.

## Introduction

Treatment of NSCLCs harboring the prevalent KRAS G12C mutation has been advanced by development of the sotorasib and adagrasib allele-selective covalent inhibitors [1–3]. In the CodeBreaK 200 phase 3 trial of patients with advanced NSCLC KRAS G12C mutant tumors, sotorasib significantly increased median progression-free survival (PFS) as compared to docetaxel (5–6 months vs 4–5 months) [4]. Moreover, in the KRYSTAL-1 phase 1–2 trial of patients with previously treated NSCLC KRAS G12C tumors, adagrasib achieved a median PFS of 6.5 months and a median overall survival (OS) of 12.6 months [1]. Despite this progress, clinical activity of these agents has been limited by acquired resistance linked to multiple genetic mechanisms that include *KRAS* secondary mutations, *MET* amplification, and activating alterations in *NRAS, BRAF* and *RET* [5–8]. Identification of KRAS switch-II pocket mutations that impair binding of sotorasib and adagrasib uncovered the potential of RAS(ON) tricomplex targeted agents, such as RMC-6236 (NCT05379985) and RMC-7977, to circumvent this resistance [5, 8–12]. Nonetheless, the pleotropic alterations converging on RAS-MAPK reactivation have limited subsequent treatment options in settings of resistance to KRAS/RAS inhibitors [5–9].

The *MUC1* gene appeared in mammals to protect barrier epithelia lining the respiratory tract from loss of homeostasis [13–15]. *MUC1* is aberrantly expressed in over 80% of NSCLCs and is associated with poor disease-free and overall survival [16, 17]. *MUC1* encodes a transmembrane MUC1-C/M1C non-mucin subunit that is activated by inflammation and promotes wound healing [15, 18]. Activation of M1C is theoretically reversible with restitution of homeostasis; however, prolonged M1C activation by chronic inflammation induces lineage plasticity and epigenetic reprogramming in association with driving malignant transformation [13–15]. Along these lines, M1C drives EMT, self-renewal capacity and tumorigenicity of NSCLC KRAS mutant cells [19, 20]. There is, however, no known involvement of M1C in resistance of NSCLC KRAS G12C mutant cells to KRAS inhibitors.

The present studies show that M1C is induced in the response of NSCLC KRAS G12C cells to sotorasib and adagrasib by an inflammatory STAT1-mediated mechanism. We also demonstrate that M1C confers resistance to KRAS G12C inhibitors by activating the NF-κB pathway with induction of EMT and mucin genes. Of translational significance, we report that a M1C ADC is effective against sotorasib-resistant NSCLC KRAS G12C cell line and patient-derived tumor xenografts. These findings identify M1C as a target for the treatment of patients with NSCLC KRAS G12C tumors refractory to KRAS inhibition.

## Methods

### Cell culture

H358 KRAS G12C mutant, TP53 null (ATCC, Manassas, VA, USA), HCC44 KRAS G12C mutant, TP53 null (ATCC) and H2122 KRAS G12C mutant, TP53 null (ATCC) cells were cultured in RPMI 1640 medium (Corning, NY, USA) supplemented with 10% FBS and 2 mM glutamine. MGH1138 (KRAS G12C, STK11 mutant) and MGH1112 (KRAS G12C, STK11 and KEAP1 mutant) cells obtained from Aaron Hata were maintained in RPMI1640 medium with 10% FBS as described [21]. Cells were treated with sotorasib and adagrasib at sub-lethal concentrations of 50 and 50–100 nM, respectively [22]. Sotorasib-resistant H358-SR cells maintained in the presence of sotorasib were grown in drug-free medium for 24 h before analysis and for 7 days during inducible M1C silencing. Authentication of the cells was performed by short tandem repeat (STR) analysis every 4 months as described [23]. Cells were monitored for mycoplasma contamination using the MycoAlert Mycoplasma Detection Kit (Lonza, Rockland, ME, USA) every 3 months.

### Gene silencing

MUC1shRNA (MISSION shRNA TRCN0000122938; Sigma) or a control scrambled shRNA (CshRNA; Sigma) was inserted into the pLKO-tet-puro vector (Plasmid #21915; Addgene, Cambridge, MA, USA). MUC1shRNA#2 (MISSION shRNA TRCN0000430218), STAT1shRNA (TRCN0000004266), NF-κBp65shRNA#1 (TRCN000014686), and NF-κBp65shRNA#2 (TRCN000014687) were produced in HEK293T cells as described [23]. Cells transduced with the vectors were selected for growth in 1–4 mg/ml puromycin as described [23]. Cells were treated with 0.1% DMSO as the vehicle control or 500 ng/ml doxycycline (DOX; Millipore Sigma).

### Quantitative reverse-transcription PCR (qRT-PCR)

Total RNA was extracted using TRIzol reagent (Thermo Fisher Scientific, Waltham, MA, USA) as described [23]. cDNA synthesis was performed with the High-Capacity RNA-to-cDNA Kit (Applied Biosystems, Grand Island, NY, USA). Quantitative PCR was performed using Power SYBR Green PCR Master Mix (Applied Biosystems) as described [23]. Primer sequences are listed in Supplementary Table S1.

### Immunoblot analysis

Total cell lysates and chromatin prepared from non-confluent cells were analyzed by immunoblotting with anti-M1C (16564, 1:1000 dilution; Cell Signaling Technology (CST), Danvers, MA, USA, RRID:AB_2798765), anti-β-actin (A5441, 1:2000 dilution; Sigma-Aldrich, Burlington, MA, USA, RRID:AB_476744), anti-Histone H3 (9715, 1:1000 dilution; CST, RRID:AB_331563), anti-pSTAT1 (9167, 1:1000 dilution; CST, RRID:AB_561284), anti-STAT1 (RAB01893, 1:500 dilution; CST, RRID:AB_3710444), anti-ERK (9107, 1:1000 dilution; CST, RRID:AB_10695739), anti-p-ERK (4377, 1:1000 dilution; CST, RRID:AB_331775), anti-NF-κB p65 (8242, 1:1000 dilution; CST, RRID:AB_10859369), anti-ZEB1 (3396, 1:1000 dilution; CST, RRID:AB_1904164), anti-E-cadherin (3195, 1:1000 dilution; CST, RRID:AB_2291471) and anti-vimentin (5741, 1:1000 dilution; CST, RRID: AB_10695459) as described [23]. Densitometric analysis of immunoblot bands was performed using Fiji/ImageJ software. Band intensities were normalized to the corresponding loading control, and relative values are presented as fold change compared with the control group.

### RNA-seq analysis

Total RNA from cells cultured separately in triplicates was isolated using Trizol reagent (Invitrogen) as described [23].

### Cell viability analyses

One to 1–2 thousand cells were seeded per well in 96-well plates (Thermo Fisher Scientific, Waltham, MA, USA) and incubated for 24 h before treatment. Cell viability was assessed using Alamar Blue staining (ThermoFisher Scientific). IC50 values were calculated by nonlinear regression of the dose response data using Prism 10.6.1 (SCR_002798, GraphPad Software). Synergistic effects were evaluated using the HSA model implemented in SynergyFinder Plus 3.0 [24]. The HSA independence model was used to determine if the drug combination effect is equal to, higher than, or lower than the expected effect (E_AB_) and thereby the combination is deemed additive (HSA = 0), synergistic (>0), or antagonistic (<0), respectively. The definition of optimal synergistic effects in vitro may not translate to in vivo models.

### Clonogenic survival assays

Cells were seeded at 2000 cells/well in 24-well plates and cultured for 24 h before treatment. The cells were stained with 0.5% crystal violet in 25% methanol on day 7–14 after treatment. Absorbance was measured at 570 nm and the results from 3 independent biological replicates reported as Relative Clonogenic Capacity as described [25].

### Tumorsphere formation assays

Cells (5 × 10^3^) were seeded per well in 6-well ultra-low attachment culture plates (Corning Life Sciences, Corning, NY, USA) and cultured as described [23]. Tumorspheres with a diameter >200 μm were counted under an inverted microscope in triplicate wells.

### Flow cytometry

Cells were harvested and incubated with either mAb 3D1 or an IgG1 kappa isotype control antibody (cat# 60070.1; STEMCELL Technologies, Vancouver, BC, Canada). Goat anti-mouse IgG (Alexa Fluor 488) (Abcam, Cambridge, MA, USA, ab150113; 1:100 dilution) was used as the secondary antibody. Data were acquired and analyzed as described [26].

### Mouse tumor model studies

Six-week-old nude female mice (The Jackson Laboratory; Bar Harbor, ME, USA) were injected subcutaneously in the flank with 2–5 × 10^6^ H358-SR or MGH1112 cells in 100 μl of a 1:1 solution of medium and Matrigel (BD Biosciences). Mice were pair-matched into treatment groups when the mean tumor volumes reached 100–150 mm^3^. Tumor measurements and body weights were recorded every 3–4 days. Mice were sacrificed when tumors reached >1000 mm^3^ as calculated by the formula: (width)^2^ x length/2. Complete responses were defined as a tumor volume <100 mm^3^ to account for residual scar tissue at the implantation sites as described [27].

### Statistical analysis

Each experiment was performed with three biological replicates. Data are expressed as the mean ± SD. The unpaired Mann–Whitney *U* test was used to determine differences between means of 2 groups. The paired *t*-test was used to compare intensities of immunoblot bands. One-way ANOVA was used for analysis of 3 and 4 groups. Asterisks represent *P ≤ 0.05, **P ≤ 0.01, ***P ≤ 0.001, ****P ≤ 0.0001 with CI = 95%.

## Results

### Targeting NSCLC KRAS G12C signaling induces M1C expression

**Fig. 1:**
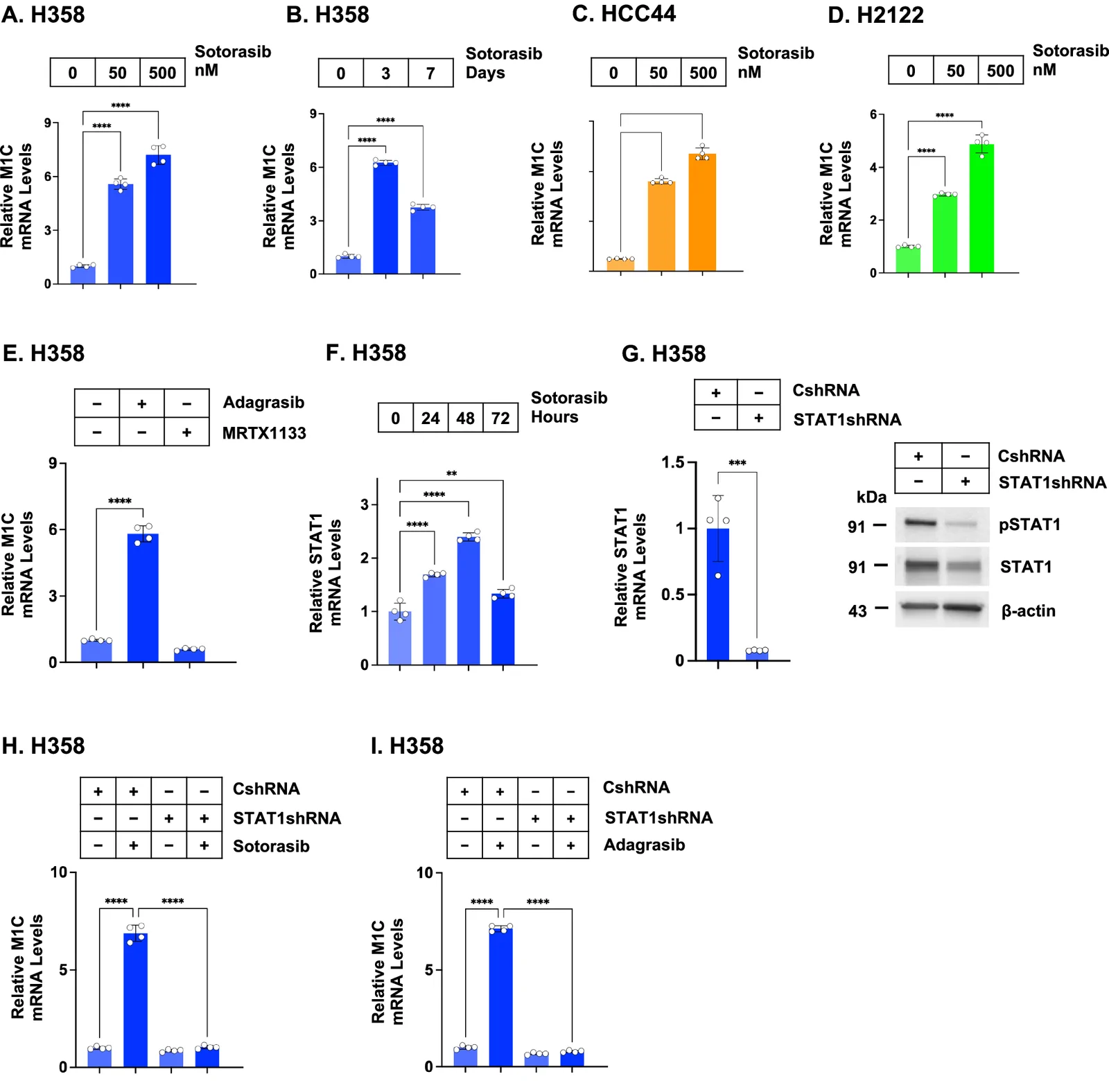
Induction of M1C expression in NSCLC KRAS G12C mutant cells treated with sotorasib and adagrasib. **A** H358 cells treated with 50 and 500 nM sotorasib for 48 h were analyzed for M1C transcripts by qRT-PCR using primers listed in Supplementary Table S1. The results (mean±SD of 4 determinations) are expressed as relative levels compared to that obtained for control cells (assigned a value of 1). **B** H358 cells treated with 50 nM sotorasib for the indicated days were analyzed for M1C transcripts. The results (mean±SD of 4 determinations) are expressed as relative levels compared to that obtained for control cells (assigned a value of 1). HCC44 (**C**) and H2122 (**D**) cells treated with 50 and 500 nM sotorasib for 48 h were analyzed for M1C transcripts. The results (mean±SD of 4 determinations) are expressed as relative levels compared to that obtained for control cells (assigned a value of 1). **E** H358 cells treated with 50 nM adagrasib and 50 nM MRTX1133 for 48 h were analyzed for M1C transcripts. The results (mean±SD of 4 determinations) are expressed as relative levels compared to that obtained for control cells (assigned a value of 1). **F** H358 cells treated with 50 nM sotorasib for the indicated times were analyzed for STAT1 transcripts. The results (mean±SD of 4 determinations) are expressed as relative levels compared to that obtained for control cells (assigned a value of 1). **G** H358/CshRNA and H358/STAT1shRNA cells were analyzed for STAT1 transcripts (left). The results (mean±SD of 4 determinations) are expressed as relative levels compared to that obtained for CshRNA cells (assigned a value of 1). Lysates were immunoblotted with antibodies against the indicated proteins (right). **H** and **I** H358/CshRNA and H358/STAT1shRNA cells treated with 50 nM sotorasib (**H**) and 50 nM adagrasib (**I**) for 48 h were analyzed for M1C transcripts. The results (mean±SD of 4 determinations) are expressed as relative levels compared to that obtained for CshRNA cells (assigned a value of 1).

*MUC1* has been linked to NSCLC progression [15]; however, there is no known involvement of the encoded M1C oncogenic subunit in resistance of NSCLC KRAS G12C mutant tumors to KRAS inhibitors. In investigating this unexplored issue, we found that treatment of H358 KRAS G12C cells with 50 and 500 nM of sotorasib for 48 h results in >5-fold increases in M1C mRNA levels (Fig. 1A). Exposure to 50 nM sotorasib further demonstrated that the induction of M1C transcripts persists through at least day 7 (Fig. 1B). In support of these results, treatment of HCC44 KRAS G12C (Fig. 1C) and H2122 KRAS G12C (Fig. 1D) cells with sotorasib was associated with comparable levels of M1C induction. We also found that treatment of H358 cells with adagrasib increases M1C expression (Fig. 1E). In regard to selectivity, PDAC KRAS G12D cells respond to the KRAS G12D inhibitor MRTX1133 with M1C induction [26], whereas MRTX1133 had little if any effect on M1C expression in H358 cells (Fig. 1E). Similar results were obtained with HCC44 (Supplementary Fig. S1A) and H2122 (Supplementary Fig. S1B) cells; that is, treatment with adagrasib, but not MRTX1133, increases M1C transcripts. In further support, treatment of NSCLC H1975 EGFR L858R/T790M cells with the mutant EGFR inhibitor osimertinib induced M1C expression; however, sotorasib had no apparent effect (Supplementary Fig. S1C). In extending these studies, H358 cells also responded to the GTP-bound KRAS G12C RMC-4998 inhibitor with increases in M1C mRNA levels (Supplementary Fig. S1D).

M1C binds directly to the STAT1 transcription factor (TF) in the response to DNA replication stress and regulates STAT1 target genes, including the transcription of *MUC1* in an auto-inductive pathway [23, 28]. Here, we found that treatment of H358 cells with sotorasib induces STAT1 transcripts (Fig. 1F), but has little effect on STAT1 protein levels (Supplementary Fig. S1E). Comparable results were obtained with adagrasib (Supplementary Figs. S1F and S1G), consistent with activation of the M1C/STAT1 transcriptional pathway [23, 28]. In support of this auto-induction, silencing STAT1 in H358 cells (Fig. 1G and Supplementary Fig. S1H) abrogated sotorasib- (Fig. 1H) and adagrasib- (Fig. 1I) induced M1C expression.

### M1C confers resistance to KRAS G12C inhibition

M1C is expressed as a ~25 kDa glycoprotein and a 17 kDa unglycosylated protein [14]. Treatment of H358 cells with sotorasib upregulated M1C protein levels (Supplementary Fig. S2A). A similar induction of the M1C protein was observed with RMC-4998 treatment (Supplementary Fig. S2A), indicating that inhibition of KRAS G12 C in the absence and presence of GTP binding increases M1C mRNA and protein levels.

**Fig. 2:**
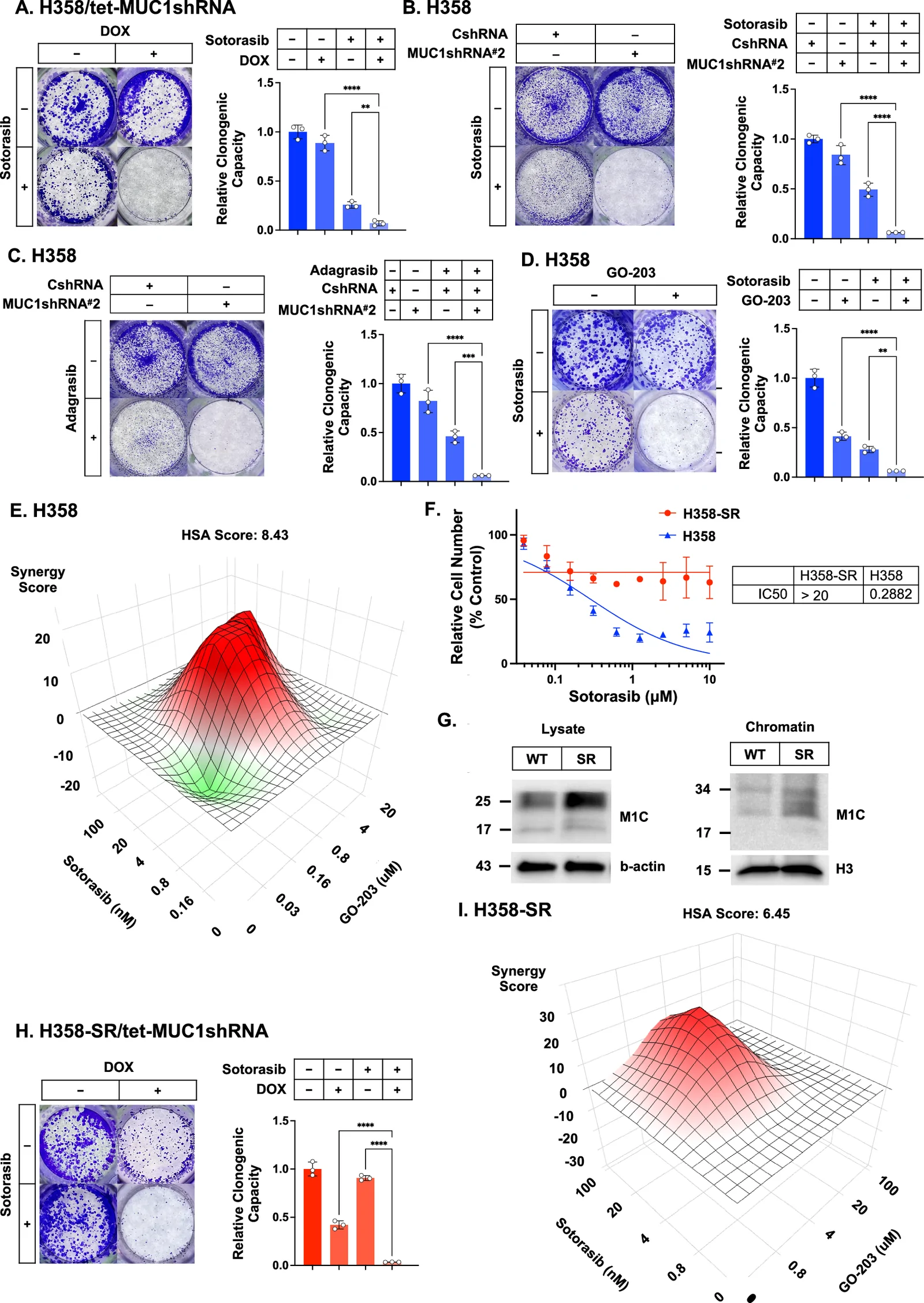
M1C is necessary for sotorasib resistance. **A** H358/tet-MUC1shRNA cells treated with vehicle or DOX for 7 days and then with 50 nM sotorasib for 7–14 days were analyzed for colony formation. Shown are representative photomicrographs of stained colonies (left). The results (mean±SD of three determinations) are expressed as relative clonogenic capacity compared to that for vehicle-treated cells (assigned a value of 1) (right). **B** and **C** H358/CshRNA and H358/MUC1shRNA#2 cells treated with 50 nM sotorasib (**B**) and 50 nM adagrasib (**C**) for 7–14 days were analyzed for colony formation. The results (mean±SD of three determinations) are expressed as relative clonogenic capacity compared to that for CshRNA cells (assigned a value of 1). **D** H358 cells treated with 0.5 μM GO-203 and 50 nM sotorasib for 7–14 days were analyzed for colony formation. The results (mean±SD of three determinations) are expressed as relative clonogenic capacity compared to that for untreated cells (assigned a value of 1). **E** H358 cells were treated with the indicated concentrations of GO-203 and sotorasib for 3 days. Cell viability was assessed using Alamar Blue staining (mean of three determinations). Indicated are the combination indices determined using HSA scores. **F** H358 and H358-SR cells treated with the indicated concentrations of sotorasib for 3 days were assessed for cell viability by Alamar Blue staining (mean of six determinations). Indicated are the IC50 values. **G** Total cell lysates (left) and chromatin (right) from H358 and H358-SR cells were immunoblotted with antibodies against the indicated proteins. **H** H358-SR/tet-MUC1shRNA cells treated with vehicle or DOX for 7 days and then with 50 nM sotorasib for 7–14 days were analyzed for colony formation. The results (mean±SD of three determinations) are expressed as relative clonogenic capacity compared to that for vehicle-treated cells (assigned a value of 1). **I** H358-SR cells were treated with the indicated concentrations of GO-203 and sotorasib for 3 days. Cell viability was assessed by Alamar Blue staining (mean of three determinations). Indicated are the combination indices determined using HSA scores.

To assess the functional significance of this response, inducible M1C silencing in H358 cells suppressed sotorasib-mediated killing as assessed by clonogenic survival (Fig. 2A and Supplementary Fig. S2B). In further evaluating this M1C dependence, we generated H358 cells stably expressing a second MUC1shRNA#2 and confirmed that downregulating M1C (Supplementary Fig. S2C) suppresses survival in response to sotorasib treatment (Fig. 2B). Analogous findings were obtained in studies with adagrasib (Fig. 2C), indicating that M1C confers resistance to these KRAS G12C inhibitors.

The M1C cytoplasmic domain contains a CQC motif that is necessary for M1C dimerization and function [29]. Targeting the M1C CQC motif with the GO-203 inhibitor enhanced sensitivity of H358 cells to sotorasib (Fig. 2D) and adagrasib (Supplementary Fig. S2D). We also found that GO-203 synergistically increases sotorasib- (Fig. 2E) and adagrasib- (Supplementary Fig. S2E) mediated killing. Whereas these results supported a role for M1C in protecting against KRAS G12C inhibitor-induced loss of survival, we generated sotorasib-resistant H358-SR cells by exposure to increasing sotorasib concentrations over 12 months (Fig. 2F). H358-SR cells also exhibited resistance to adagrasib (Supplementary Fig. S2F). M1C localizes to chromatin, where it interacts with TFs and effectors of the epigenome in regulating gene expression [15]. Analysis of H358-SR cells demonstrated upregulation of M1C levels in total lysates, as well as in chromatin, compared to parental H358 cells (Fig. 2G). Treatment of H358-SR/tet-MUC1shRNA cells with DOX suppressed M1C expression (Supplementary Fig. S2G) and reversed sotorasib resistance (Fig. 2H). In addition, GO-203 synergistically increased sotorasib sensitivity of H358-SR cells (Fig. 2I).

As a second model, we turned to H2122 KRAS G12C mutant cells that are sensitive to sotorasib (IC50 = 0.7 μM) and generated sotorasib-resistant H2122-SR cells (IC50 = 16 μM; Supplementary Fig. S2H). Analysis of H2122-SR vs H2122 cells demonstrated upregulation of M1C expression (Supplementary Fig. S2I). Moreover, targeting M1C in H2122-SR cells with GO-203 synergistically increased sotorasib killing (Supplementary Fig. S2J), confirming that M1C is necessary for the sotorasib-resistant phenotype.

### M1C induces EMT, mucin genes and sotorasib resistance

**Fig. 3:**
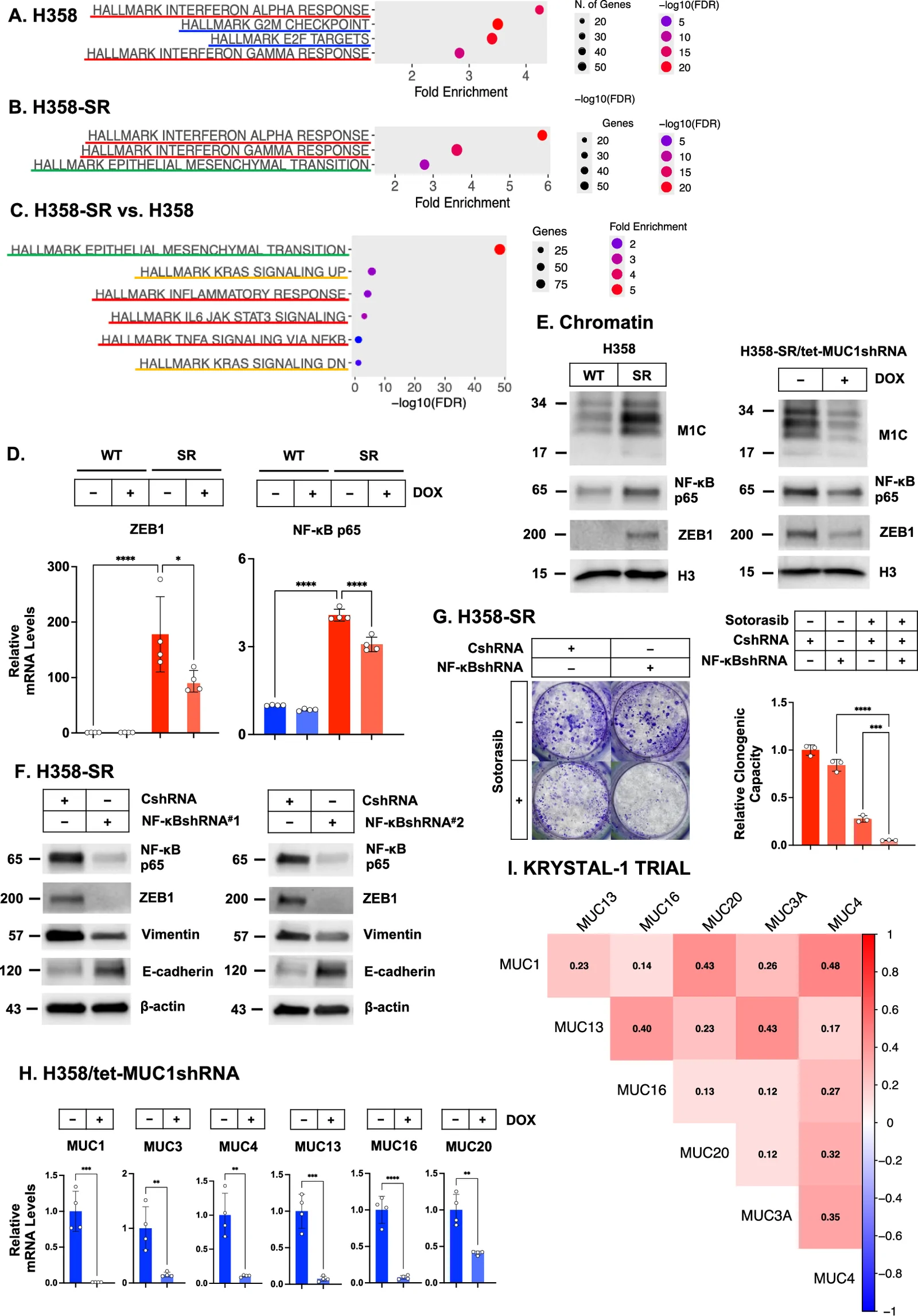
M1C drives sotorasib resistance by a NF-κB/ZEB1/EMT-dependent mechanism. **A** and **B** M1C-dependent activation of the indicated HALLMARK gene signatures in H358 (**A**) and H358-SR (**B**) cells. **C** Comparison of HALLMARK gene signatures in H358-SR vs H358 cells. **D** H358/tet-MUC1shRNA and H358-SR/tet-MUC1shRNA cells treated with vehicle or DOX for 7 days were analyzed for NF-κB and ZEB1 transcripts. The results (mean ± SD of 4 determinations) are expressed as relative levels compared to that obtained for control H358 cells (assigned a value of 1). **E** Chromatin from H358 and H358-SR cells (left) and H358-SR/tet-MUC1shRNA cells treated with vehicle or DOX for 7 days (right) was immunoblotted with antibodies against the indicated proteins. **F** Lysates from H358-SR/CshRNA, H358-SR/NF-κBshRNA#1 and H358-SR/NF-κBshRNA#2 cells were immunoblotted with antibodies against the indicated proteins. **G** H358-SR/CshRNA and H358-SR/NF-κBshRNA cells were treated with 50 nM sotorasib for 7–14 days and analyzed for colony formation. The results (mean±SD of three determinations) are expressed as relative clonogenic capacity compared to that for CshRNA cells (assigned a value of 1). **H** H358/tet-MUC1shRNA cells treated with vehicle or DOX for 7 days were analyzed for the indicated mucin gene transcripts. The results (mean±SD of 4 determinations) are expressed as relative levels compared to that obtained for control H358 cells (assigned a value of 1). **I** Correlations of mucin gene expression from analysis of the KRYSTAL-1 trial dataset.

To identify M1C-dependent mechanisms that confer sotorasib resistance, we compared the transcriptomes of parental H358 and H358-SR cells. Silencing M1C in H358 and H358-SR cells downregulated 765 and 206 genes, respectively (Supplementary Figs. S3A–S3C). Among these, we identified 102 genes that included interferon stimulated genes (ISGs), such as IFIT1 and MX1/2, among others (Supplementary Fig. S3C). GSEA of the parental H358 cell RNA-seq dataset uncovered significant M1C-dependent activation of the (i) HALLMARK INTERFERON ALPHA and GAMMA, and (ii) HALLMARK G2M CHECKPOINT, E2F TARGETS and MITOTIC SPINDLE signatures (Fig. 3A), indicative of driving inflammatory and proliferative pathways. Analysis of the H358-SR RNA-seq data further demonstrated that M1C significantly activates the HALLMARK INTERFERON ALPHA, but not the G2M CHECKPOINT, E2F TARGETS and MITOTIC SPINDLE, gene signatures (Fig. 3B, C).

The ZEB1 EMT TF drives a mesenchymal phenotype in carcinoma cells [30]. M1C binds directly to NF-κB p65 (RELA) and contributes to the regulation of NF-κB p65 target genes, including MUC1 in an auto-inductive pathway and ZEB1 [31, 32]. M1C also binds directly to ZEB1 in driving EMT [32, 33]. Analysis of H358-SR vs H358 cells demonstrated M1C-dependent increases in NF-κB p65 and ZEB1 mRNA levels (Fig. 3D). We also found that NF-κB and ZEB1 proteins are increased in chromatin from H358-SR vs H358 cells and that silencing M1C downregulates their expression (Fig. 3E).

To assess functional involvement of the M1C→NF-κB→ZEB1 pathway, we silenced NF-κB in H358-SR cells with different shRNAs and found (i) downregulation of ZEB1 and (ii) suppression of the EMT phenotype, as evidenced by upregulation of E-cadherin and decreases in vimentin (Fig. 3F). Moreover, as shown for M1C, silencing NF-κB reversed resistance of H358-SR cells to sotorasib (Fig. 3G).

These results were extended with studies of H2122-SR cells, which demonstrated upregulation of M1C, NF-κB and ZEB1 in chromatin, as compared to parental H2122 cells (Supplementary Fig. S3G). Moreover, treatment of H2122-SR cells with GO-203 suppressed NF-κB and ZEB1 chromatin levels (Supplementary Fig. S3H). These findings indicate that the M1C→NF-κB→ZEB1 pathway drives EMT in association with sotorasib resistance.

RAS inhibitor resistance in mouse KL2 Kras G12C tumors has been linked to the induction of a mucinous gene program [5]. Silencing M1C in H358 cells decreased expression of the (i) secreted MUC3A, and (ii) transmembrane MUC4, MUC13, MUC16 and MUC20 mucins (Fig. 3H). Consistent with these results, analysis of the KRYSTAL-1 trial dataset from patients with previously treated NSCLC KRAS G12C tumors demonstrated that MUC1 significantly associates with MUC3A, MUC4, MUC13, MUC16 and MUC20 expression, indicating that M1C is master regulator of these mucin genes (Fig. 3I). Mucins activate the NF-κB pathway, which drives mucin gene expression in auto-inductive pathways [34–37]. Noteworthy is that these mucins also promote EMT [38], indicating that the M1C→NF-κB pathway integrates induction the mucin gene program with EMT and drug resistance.

### M1C promotes resistance of patient-derived NSCLC KRAS G12C cells to KRAS inhibition

**Fig. 4:**
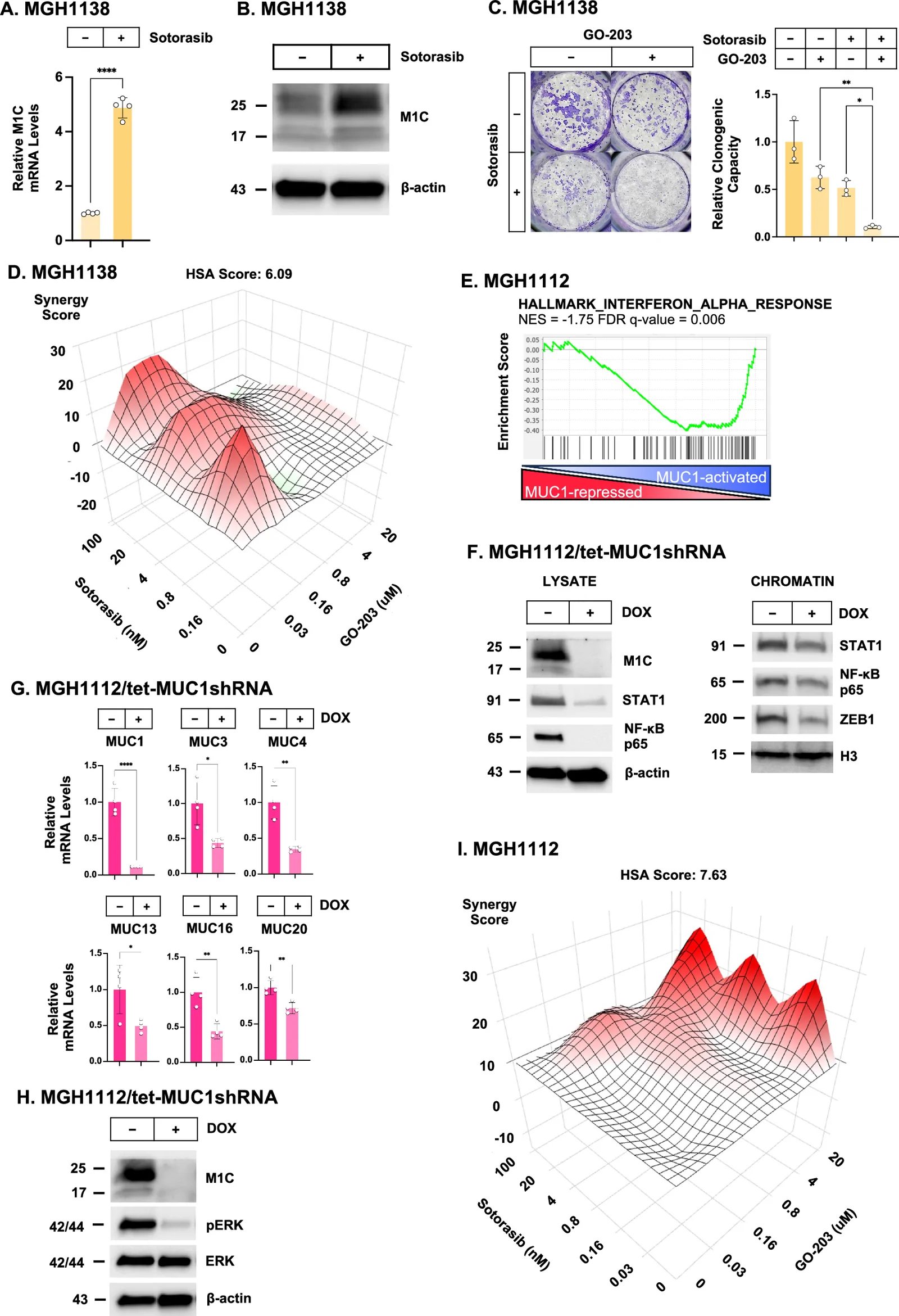
Patient-derived MGH1138 and MGH1112 KRAS G12C cells are M1C-dependent. **A** and **B** MGH1138 cells treated with 50 nM sotorasib for 48 h were analyzed for M1C transcripts. The results (mean ± SD of 4 determinations) are expressed as relative levels compared to that obtained for control cells (assigned a value of 1) (**A**). Lysates were immunoblotted with antibodies against the indicated proteins (**B**). **C** MGH1138 cells treated with 0.5 μM GO-203 and 50 nM sotorasib for 7–14 days were analyzed for colony formation. The results (mean±SD of three determinations) are expressed as relative clonogenic capacity compared to that for untreated cells (assigned a value of 1). **D** MGH1138 cells were treated with the indicated concentrations of GO-203 and sotorasib for 3 days. Cell viability was assessed by Alamar Blue staining (mean of three determinations). Indicated are the combination indices determined using HSA scores. **E** GSEA of RNA-seq data from MGH1112 cells with M1C silencing using the HALLMARK INTERFERON ALPHA RESPONSE gene signature. **F** Cell lysates and chromatin from MGH1112/tet-MUC1shRNA cells treated with vehicle or DOX for 7 days were immunoblotted with antibodies against the indicated proteins. **G** MGH1112/tet-MUC1shRNA cells treated with vehicle or DOX for 7 days were analyzed for the indicated mucin gene transcripts. The results (mean±SD of 4 determinations) are expressed as relative levels compared to that obtained for vehicle-treated cells (assigned a value of 1). **H** Lysates from MGH1112/tet-MUC1shRNA cells treated with vehicle or DOX for 7 days were immunoblotted with antibodies against the indicated proteins. **I** MGH1112 cells were treated with the indicated concentrations of GO-203 and sotorasib for 3 days. Cell viability was assessed by Alamar Blue staining (mean of three determinations). Indicated are the combination indices determined using HSA scores.

To extend these results, we studied patient-derived NSCLC KRAS G12C mutant MGH1138 cells, which like H358 cells are sensitive to sotorasib [21]. As found in the H358 and H2122 cell line models, treatment of MGH1138 cells with sotorasib was associated with induction of M1C mRNA (Fig. 4A) and protein (Fig. 4B) levels. Similar results were obtained when treating MGH1138 cells with adagrasib (Supplementary Fig. S4A) and RMC-4998 (Supplementary Fig. S4B). Targeting M1C in MGH1138 cells with GO-203 increased sensitivity to sotorasib as evidenced by significant decreases in clonogenic survival with the combination as compared to that with either agent alone (Fig. 4C). In accordance with these results, GO-203 treatment was synergistic with sotorasib in suppressing MGH1138 cell survival (Fig. 4D).

As a second NSCLC patient-derived model, we studied MGH1112 KRAS G12C cells that, like H358-SR cells, are resistant to sotorasib with an IC50 > 1 μM [21].

GSEA of RNA-seq data from MGH1112 cells revealed that, as shown for H358-SR cells, silencing M1C significantly suppresses activation of the HALLMARK INTERFERON ALPHA gene signature (Fig. 4E). As also found with H358-SR cells, silencing M1C was associated with upregulation of the HALLMARK E2F CHECKPOINT (Supplementary Fig. S4C) and HALLMARK G2M CHECKPOINT (Supplementary Fig. S4D) signatures. These results in H358-SR and MGH1112 cells indicate that M1C drives inflammatory IFN signaling, while suppressing proliferative pathways, in the sotorasib-resistant phenotype.

As additional parallels with H358-SR cells, silencing M1C in MGH1112 cells reduced expression of STAT1, NF-κB and ZEB1 (Fig. 4F and Supplementary Figs. S4E and S4F) and mucin genes (Fig. 4G). Moreover, targeting M1C in MGH1112 cells downregulated the feedback pERK pathway (Fig. 4H and Supplementary Fig. S4G) and was synergistic with sotorasib in suppressing survival (Fig. 4I). These results provide further support for involvement of M1C in integrating induction of the EMT/mucin phenotype with sotorasib resistance.

### Targeting M1C in sotorasib-resistant NSCLC KRAS G12C cell lines with an antibody-drug conjugate

**Fig. 5:**
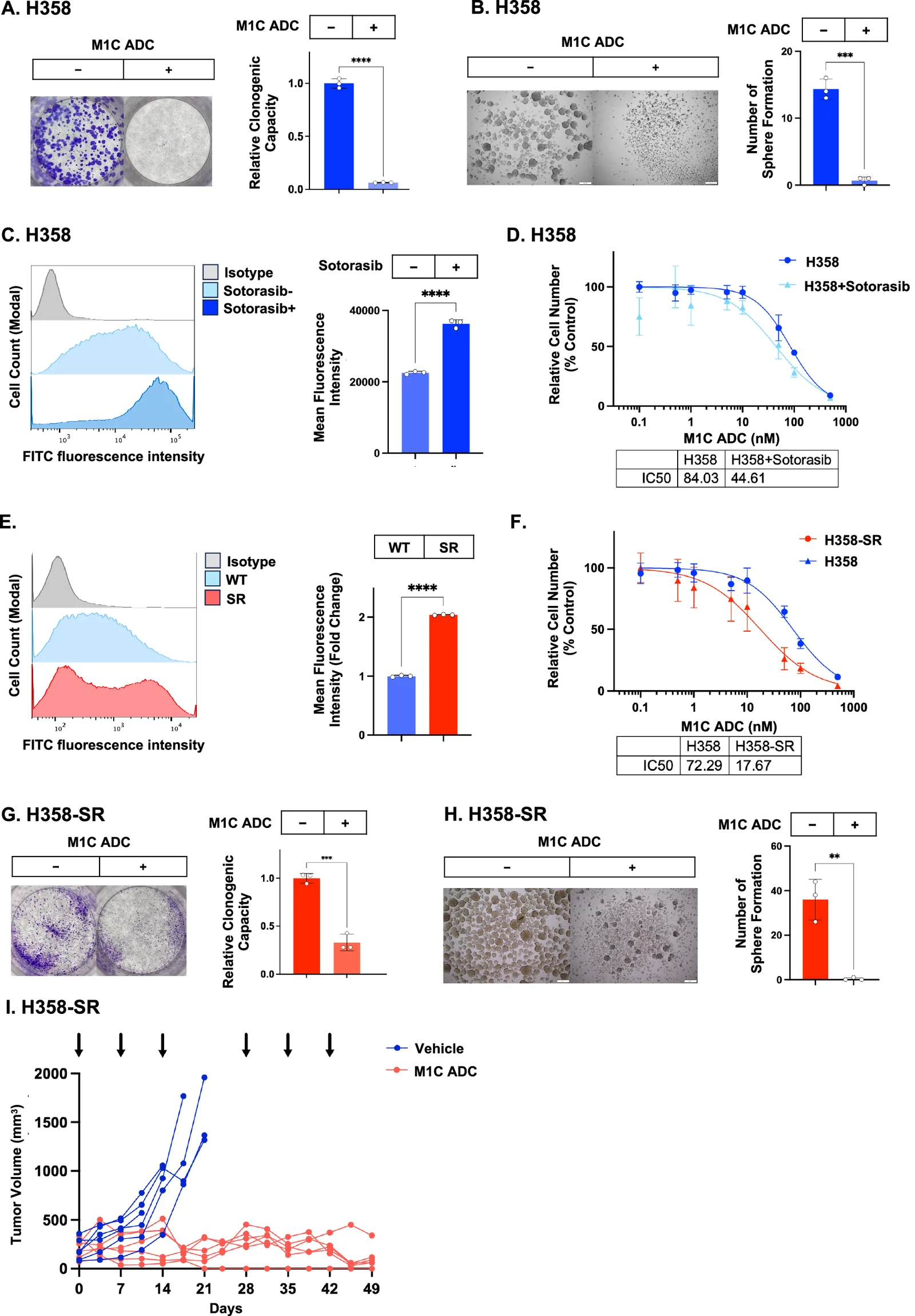
Targeting M1C with an ADC is effective against sotorasib-resistant NSCLC KRAS G12C mutant cells. **A** H358 cells treated with 20 nM M1C ADC for 7–14 days were analyzed for colony formation. Shown are representative photomicrographs of stained colonies (left). The results (mean±SD of three determinations) are expressed as relative clonogenic capacity compared to that for untreated cells (assigned a value of 1) (right). **B** H358 cells treated with vehicle or 50 nM M1C ADC for 7 days were analyzed for tumorsphere formation. Shown are representative photomicrographs of tumorspheres (left). The results (mean±SD of three determinations) are expressed as number of tumorsphere formations compared to that for vehicle treated cells (right). **C** H358 cells left untreated or treated with 50 nM sotorasib for 2 days were analyzed by flow cytometry with a control IgG and anti-M1C. Shown are histograms of cells positive for M1C expression (left). The bar plot shows the mean fluorescence intensity (MFI) presented as the mean ± SD of three independent determinations (right). **D** H358 cells treated with vehicle or 50 nM sotorasib for 2 days and then M1C ADC for 7 days were analyzed for cell viability by Alamar blue staining. **E** H358 and H358-SR cells were analyzed by flow cytometry with a control IgG and anti-M1C. Shown are histograms of cells positive for M1C expression (left). The bar plot shows the MFI, presented as the mean ± SD of three independent determinations (right). **F** H358 and H358-SR cells treated with the indicated concentrations of M1C ADC for 7 days were analyzed for cell viability by Alamar Blue staining. The results (mean±SD of six determinations) are expressed as relative cell number (% control) compared to that for untreated cells. Indicated are the M1C ADC IC50 values. **G** H358-SR cells treated with 50 nM M1C ADC for 7–14 days were analyzed for colony formation. Shown are representative photomicrographs of stained colonies (left). The results (mean±SD of three determinations) are expressed as relative clonogenic capacity compared to that for untreated cells (assigned a value of 1) (right). **H** H358-SR cells treated with vehicle or 50 nM M1C ADC for 7 days were analyzed for tumorsphere formation. Shown are representative photomicrographs of tumorspheres (left). The results (mean±SD of three determinations) are expressed as number of tumorsphere formations compared to that for vehicle treated cells (assigned a value of 1) (right). **I** Nude mice were injected subcutaneously with 5 × 10^6^ H358-SR cells. Mice were randomized into two groups when the mean tumor volume reached 100 mm^3^ and then treated with vehicle (*n* = 6) or 7.5 mg/kg M1C ADC weekly × 3 every 28 days for 2 cycles (*n* = 6).

Having found that sotorasib-resistant NSCLC KRAS G12C cells are M1C-dependent, we asked if M1C is a potential target for their treatment. To this end, we assessed the effects of a M1C ADC generated with an antibody against the M1C extracellular domain and conjugated it with a cleavable linker to the MMAE payload [39]. The M1C ADC is (i) inactive against tumor cells null for M1C expression [39], and (ii) effective against M1C-positive PDAC KRAS G12D cells resistant to MRTX1133 [26]. However, there is no information in regard to activity of the M1C ADC in NSCLC KRAS G12C cell models and especially those resistant to sotorasib. Here, we found that H358, HCC44 and H2122 cells are sensitive to the M1C ADC with IC50 values 84, 62 and 21 nM, respectively (Supplementary Fig. S5A). M1C ADC treatment of H358 cells inhibited clonogenic survival (Fig. 5A) and tumorsphere formation (Fig. 5B). Similar effects were observed with H2122 cells (Supplementary Figs. S5B and S5C), indicating that the M1C ADC is effective in suppressing self-renewal capacity.

Consistent with the demonstration that sotorasib induces M1C expression, we found that sotorasib and adagrasib increase M1C levels on the surface of H358 cells (Fig. 5C and Supplementary Fig. S5D) and sensitivity to the M1C ADC (Fig. 5D and Supplementary Fig. S5E). Interestingly, we also found that (i) M1C expression is significantly increased on the surface of H358-SR vs H358 cells (Fig. 5E), and (ii) H358-SR cells are more sensitive to M1C ADC treatment (IC50 = 18 nM) than H358 cells (IC50 = 72 nM) (Fig. 5F). The M1C ADC was also more effective against H2122-SR vs H2122 cells (Supplementary Fig. S5F), indicating that sotorasib resistance is associated with increased sensitivity to this ADC.

These results were extended by the demonstration that the M1C ADC suppresses H358-SR (Fig. 5G) and H2122-SR (Supplementary Fig. S5G) clonogenic survival. The M1C ADC was also highly effective in inhibiting H358-SR (Fig. 5H) and H2122-SR (Supplementary Fig. S5A) tumorsphere formation. Given these results, we treated established H358-SR tumor xenografts with the M1C ADC and found induction of complete responses in 4 of 6 mice (Fig. 5I) in the absence of weight loss or other overt toxicities (Supplementary Fig. S5I). Of note, studies of H358-SR cells grown in the absence of sotorasib demonstrate that the resistant phenotype persists for at least 30 days (Supplementary Fig. S5J).

### M1C is a potential target for ADC treatment of patients with NSCLC KRAS G12C tumors resistant to KRAS inhibitors

**Fig. 6:**
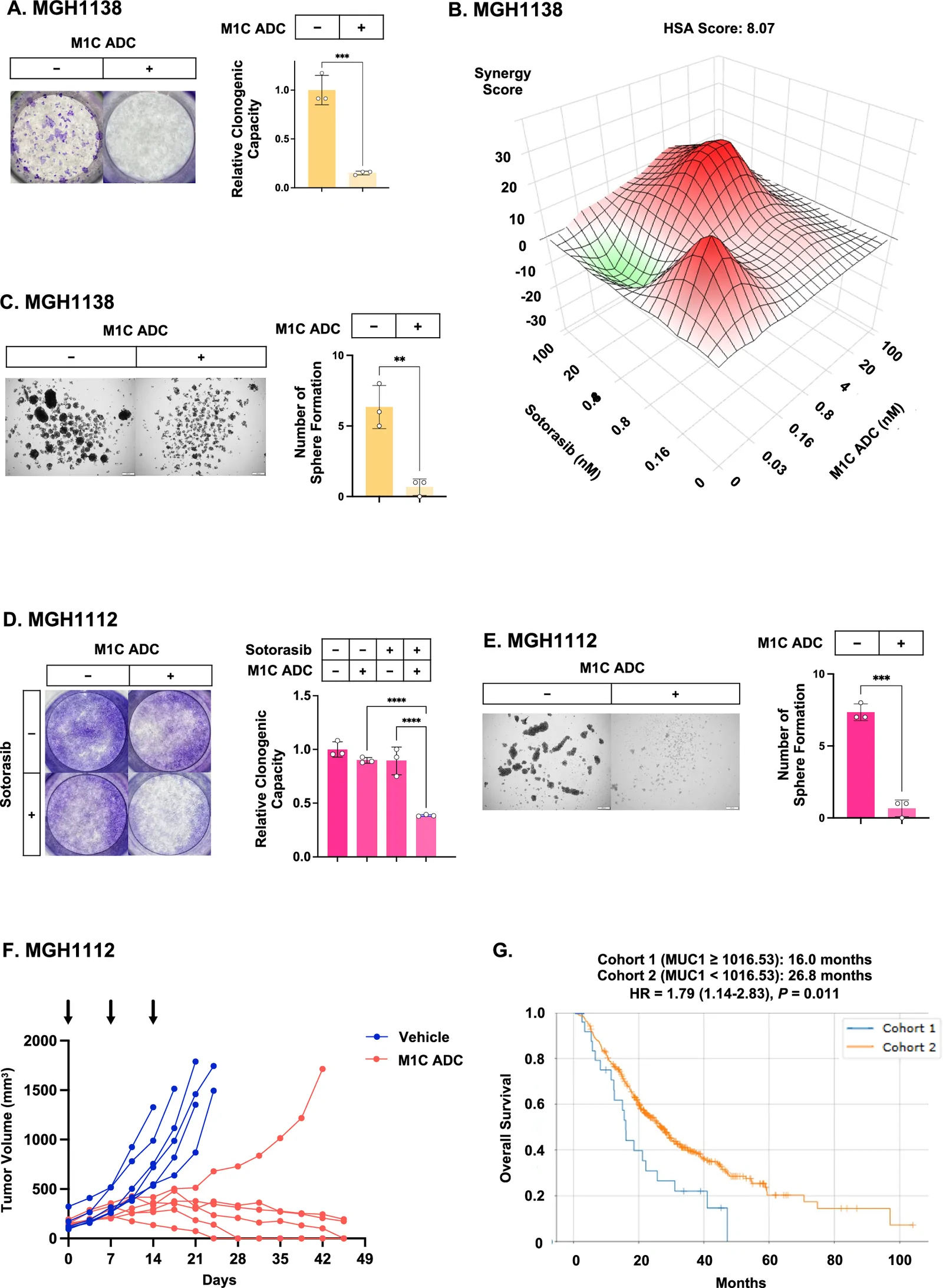
M1C is a potential target for the treatment of human NSCLC KRAS G12C tumors with the M1C ADC. **A** MGH1138 cells treated with 50 nM M1C ADC for 7 days were analyzed for colony formation. Shown are representative photomicrographs of stained colonies (left). The results (mean±SD of three determinations) are expressed as relative clonogenic capacity compared to that for untreated cells (assigned a value of 1) (right). **B** MGH1138 cells were treated with the indicated concentrations of M1C ADC and sotorasib for 7 days. Cell viability was assessed by Alamar Blue staining (mean of three determinations). Indicated are the combination indices determined using HSA scores. **C** MGH1138 cells treated with vehicle or 50 nM M1C ADC for 7 days were analyzed for tumorsphere formation. Shown are representative photomicrographs of tumorspheres (left). The results (mean ± SD of three determinations) are expressed as number of tumorsphere formations compared to that for vehicle treated cells (right). **D** MGH1112 cells treated with vehicle or 0.5 μM sotorasib and 5 nM M1C -ADC for 7–14 days were analyzed for colony formation. Shown are representative photomicrographs of stained colonies (left). The results (mean±SD of three determinations) are expressed as relative clonogenic capacity compared to that for untreated cells (assigned a value of 1) (right). **E** MGH1112 cells treated with vehicle or 50 nM M1C ADC for 7 days were analyzed for tumorsphere formation. Shown are representative photomicrographs of tumorspheres (left). The results (mean±SD of three determinations) are expressed as number of tumorsphere formations compared to that for vehicle treated cells (right). **F** Nude mice were injected subcutaneously with 5 × 10^6^ MGH1112 cells. Mice were randomized into two groups when the mean tumor volume reached 100 mm^3^ and then treated with vehicle (*n* = 6) or 7.5 mg/kg M1C ADC weekly × 3 (1 cycle) (*n* = 6). **G** Overall survival for patients with NSCLC G12C mutant tumors treated with sotorasib and/or adagrasib according to MUC1 high (cohort 1) and MUC1 low (cohort 2) levels using the CARIS CODEai database.

In extending these results with patient-derived cells, we found that M1C ADC treatment of MGH1138 cells is effective in suppressing clonogenic survival (Fig. 6A). Additionally, treatment of MGH1138 cells with the M1C ADC in combination with sotorasib demonstrated synergistic activity (Fig. 6B). We also found that exposure of MGH1138 cells to the M1C ADC is effective in inhibiting tumorsphere formation (Fig. 6C), indicating that the ADC could be used alone and in combination with sotorasib to suppress self-renewal capacity.

In studies of sotorasib-resistant MGH1112 cells, the M1C ADC was active in suppressing clonogenic survival (Supplementary Fig. S6A). In addition, the M1C ADC was more effective in combination with sotorasib than either agent alone (Fig. 6D and Supplementary Fig. S6B). The M1C ADC was also highly effective in inhibiting MGH1112 self-renewal capacity as evidenced by suppression of tumorsphere formation (Fig. 6E). In addition, M1C ADC treatment of 6 mice with established MGH1112 tumor xenografts resulted in 3 with complete responses and 2 with stable disease (Fig. 6F) in the absence of significant weight loss (Supplementary Fig. S6C).

In support of M1C as a potential clinical target for the ADC, analysis of NSCLC KRAS G12C tumors from patients treated with sotorasib and/or adagrasib in the CARIS CODEai database demonstrated that MUC1 high vs low expression levels associate with a significant decrease in overall survival (Fig. 6G).

## Discussion

**Fig. 7:**
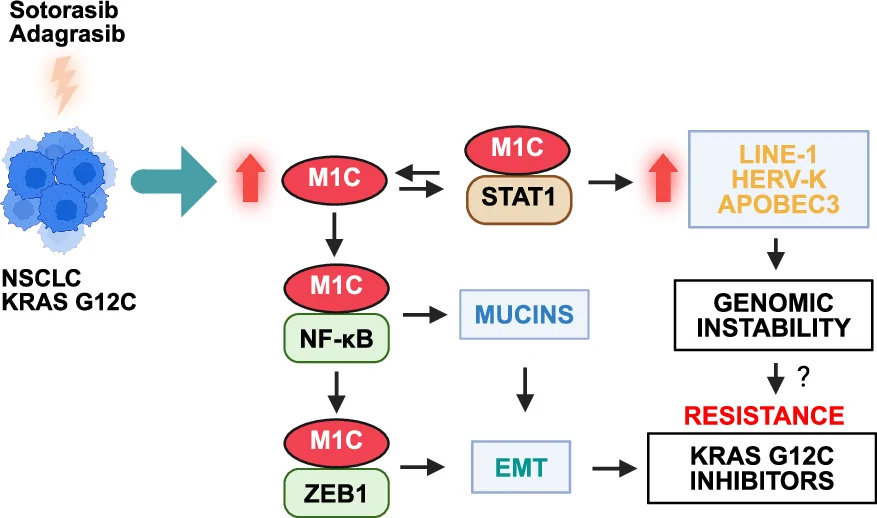
Schema depicting involvement of M1C in conferring resistance of NSCLC KRAS G12C cells to KRAS inhibition. Treatment of NSCLC KRAS G12C cells with sotorasib or adagrasib induces M1C expression by activation of the M1C/STAT1 auto-inductive pathway. M1C/STAT1 signaling activates the (i) LINE-1 and HERV retrotransposons, and (ii) APOBEC3 expression, which collectively drive genomic instability and drug resistance [15]. M1C is also induced by the RAS(ON) inhibitor RMC-7977, but not MRTX1133, indicating that this represents a response to KRASG12C/RAS inhibition. Upregulation of M1C activates NF-κB p65 by a direct interaction and thereby regulates NF-κB target genes, including MUC1 in an auto-inductive pathway [15]. M1C/NF-κB signaling induces the downstream ZEB1 EMT TF and mucin genes that drive EMT and drug resistance. In this way, M1C activates the STAT1 and NF-κB pathways in conferring resistance to KRAS G12C inhibition by epigenetically integrating chronic inflammation with the induction of EMT.

Acquired resistance to sotorasib and adagrasib has been linked to diverse genetic alterations [5–9, 39]. The present results uncover a non-genetic mechanism in which M1C is activated in the response of NSCLC KRAS G12C mutant cells to treatment with these selective KRAS G12C (OFF) inhibitors (Fig. 7). M1C interacts with RTKs at the NSCLC cell membrane and functions as a scaffold for transducing downstream signals [14, 15]. In this way, the M1C cytoplasmic domain forms complexes with SHP2, PI3K, SHC, PLCg and GRB2/SOS that integrate RTK and RAS signaling pathways [14, 15]. Interestingly, this induction of M1C is not restricted to inhibiting inactive KRAS G12C, as it extends to targeting GTP-bound KRAS G12C with the RMC-4998 inhibitor. Studies in PDAC cells have further demonstrated that M1C is induced by the KRAS G12D MRTX1133 inhibitor [26], indicating that M1C is a shared effector of resistance to distinct allele-selective KRAS inhibitors across different cancer cell types. Identification of this novel M1C function in the KRAS inhibitor field supports further investigation with pan-RAS targeted agents, such as RMC-6236.

In addressing how M1C plays a role in KRAS inhibitor resistance, M1C evolved in mammals to protect barrier tissues from loss of homeostasis [14, 15]. M1C functions as a scaffold in integrating the proliferative RTK/RAS axis with induction of inflammatory pathways that drive wound repair [14, 15]. As a result, the *MUC1* gene is activated by an inflammatory memory response to DNA replication stress induced by targeted agents [23, 40]. Conversely, as an adverse maladaptation, prolonged M1C activation by chronic inflammation promotes malignant progression [14, 15]. Our findings demonstrate that inhibition of KRAS G12C signaling induces M1C by a STAT1-dependent pathway (Fig. 7), which has been linked to activation of (i) IFN type I/II signaling, (ii) LINE-1 and HERV transposons, (iii) APOBEC3 and other ISGs, (iv) inflammatory memory, and (v) drug resistance [23, 40–42].

The finding that M1C is activated by KRAS G12C inhibitors invoked the possibility that upregulation of M1C may be responsible for the drug-resistant phenotype. The STAT and NF-κB pathways intersect in complex regulatory networks in association with driving chronic inflammation in cancer [43]. In KRAS G12C inhibitor-resistant cell models, we found that M1C, as well as NF-κB and ZEB1, levels are upregulated in chromatin. Like STAT1, M1C forms a direct complex with NF-κB and regulates NF-κB target genes [31, 33]. As a result, M1C/NF-κB complexes induce *ZEB1* transcription and, in turn, M1C directly activates ZEB1 [32]. Here, we found that the M1C/NF-κB pathway is responsible for progression of wild-type NSCLC KRAS G12C mutant cells to the KRAS inhibitor-resistant phenotype by driving ZEB1 and the EMT state (Fig. 7). These findings support a model in which (i) M1C is induced by STAT1 in sotorasib-treated parental cells, and (ii) M1C→NF-κB→ZEB1 signaling drives EMT and resistance to KRAS G12C inhibition (Fig. 7). Our results linking M1C-driven EMT with KRAS inhibitor resistance lend mechanistic support to previous work implicating the importance of this association [44].

The M1C cytoplasmic domain activates JAK1-STAT1 [28], TAK1-NF-κB [31, 45] and ZEB1 [32] signaling through similar binding regions, indicating that their induction is conferred by integrated M1C-dependent inflammatory mechanisms [14, 15]. Our results further demonstrate that M1C is necessary for the induction of mucin genes, which promote EMT and thereby drug resistance [38]. By extension and in support of those findings, analysis of the KRYSTAL-1 trial dataset from patients with previously treated NSCLC KRAS G12C tumors demonstrated that MUC1 significantly associates mucin gene expression. In this regard, M1C drives EMT and drug resistance by integrating NF-κB-mediated induction of ZEB1 and mucin expression (Fig. 7). Additional correlative studies will be needed when datasets become available from patients with NSCLC KRAS G12C mutant tumors resistant to sotorasib.

Patients with NSCLC KRAS G12C tumors resistant to sotorasib and/or adagrasib have few treatment options [6–9]. Having identified M1C is a potential target for the treatment of cells resistant to KRAS G12C inhibitors, we asked if a M1C ADC is effective in this setting. In this regard, although the ADC is active against PDAC KRAS G12D cells resistant to MRTX1133 [26], there was no a priori reasoning this would extend to NSCLC KRAS G12C cells with resistance to sotorasib. The present results demonstrate that the M1C ADC is active in vitro against NSCLC KRAS G12C parental, as well as sotorasib-resistant, cells. In addition, the M1C ADC was highly effective against H358-SR and patient-derived sotorasib-resistant MGH1112 xenograft tumors. Of further relevance as a potential target, we found that upregulation of MUC1 expression in NSCLC KRAS G12C tumors associates with decreases in overall survival of patients treated with sotorasib/adagrasib.

Our findings collectively lend support for development of the M1C ADC for treatment of sotorasib/adagrasib-resistant tumors. The M1C ADC has also been shown to be effective against drug-resistant (i) HR+/HER2− breast [46], (ii) CRPC/NEPC prostate [47] and (iii) PDAC KRAS G12D [26] tumor models, indicating that this agent may be broadly active in settings of tumors unresponsive to targeted agents. Along these lines, the M1C ADC is under development by the NCI NExT Program for IND-enabling studies and early phase clinical evaluation. If well-tolerated in the clinic, the present results could provide support for assessing efficacy of the M1C ADC as a single agent in patients with NSCLC KRAS G12C tumors resistant to sotorasib/adagrasib. Additional preclinical studies of the M1C ADC in combinations with sotorasib/adagrasib will require more detailed experimentation of dosing, tolerability and response.

In summary, the present results demonstrate that M1C is necessary for KRAS G12C inhibitor resistance and is a potential target for treatment of patients with refractory NSCLC KRAS G12C tumors.

## Supplementary information

Supplementary Figure 1

Supplementary Figure 2

Supplementary Figure 3

Supplementary Figure 4

Supplementary Figure 5

Supplementary Figure 6

Supplementary Figure Legends

Supplementary Table S1

## Data Availability

The RNA-seq data have been deposited in the Gene Expression Omnibus under accession numbers GSE315052, GSE315054, and GSE324028. Additional raw data is available from the corresponding author upon reasonable request.

## Materials availability

Materials described here, including raw source data, will be made freely available to researchers for non-commercial purposes.
