# Supplementary material for: M1C is a druggable target for NSCLC KRAS G12C mutant tumors resistant to KRAS inhibitors
Source: Oncogene. 2026 Aug 5;45(38):4088–100. doi: 10.1038/s41388-026-03934-2 (PMC13577901; doi:10.1038/s41388-026-03934-2)
Supplement: Supplementary file 1 [file 41388_2026_3934_MOESM1_ESM.pdf]

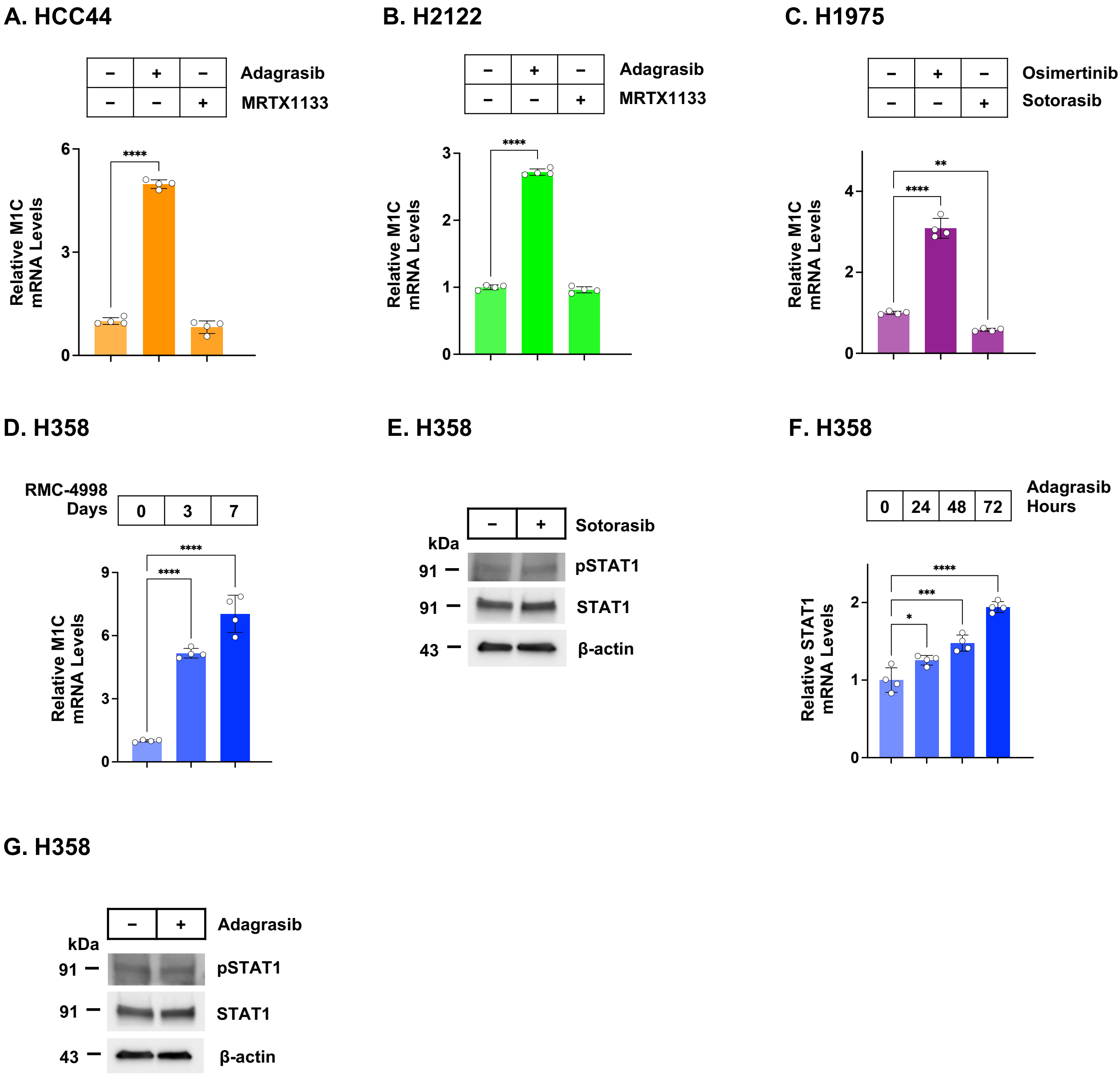
A. HCC44
Adagrasib
MRTX1133
Relative M1C mRNA Levels
****
B. H2122
Adagrasib
MRTX1133
Relative M1C mRNA Levels
****
C. H1975
Osimertinib
Sotorasib
Relative M1C mRNA Levels
****
**
D. H358
RMC-4998 Days
0
3
7
Relative M1C mRNA Levels
****
E. H358
Sotorasib
kDa
91
91
43
pSTAT1
STAT1
β-actin
F. H358
Adagrasib Hours
0
24
48
72
Relative STAT1 mRNA Levels
****
***
*
G. H358
Adagrasib
kDa
91
91
43
pSTAT1
STAT1
β-actin
