# Supplementary material for: M1C is a druggable target for NSCLC KRAS G12C mutant tumors resistant to KRAS inhibitors
Source: Oncogene. 2026 Aug 5;45(38):4088–100. doi: 10.1038/s41388-026-03934-2 (PMC13577901; doi:10.1038/s41388-026-03934-2)
Supplement: Supplementary file 2 [file 41388_2026_3934_MOESM2_ESM.pdf]

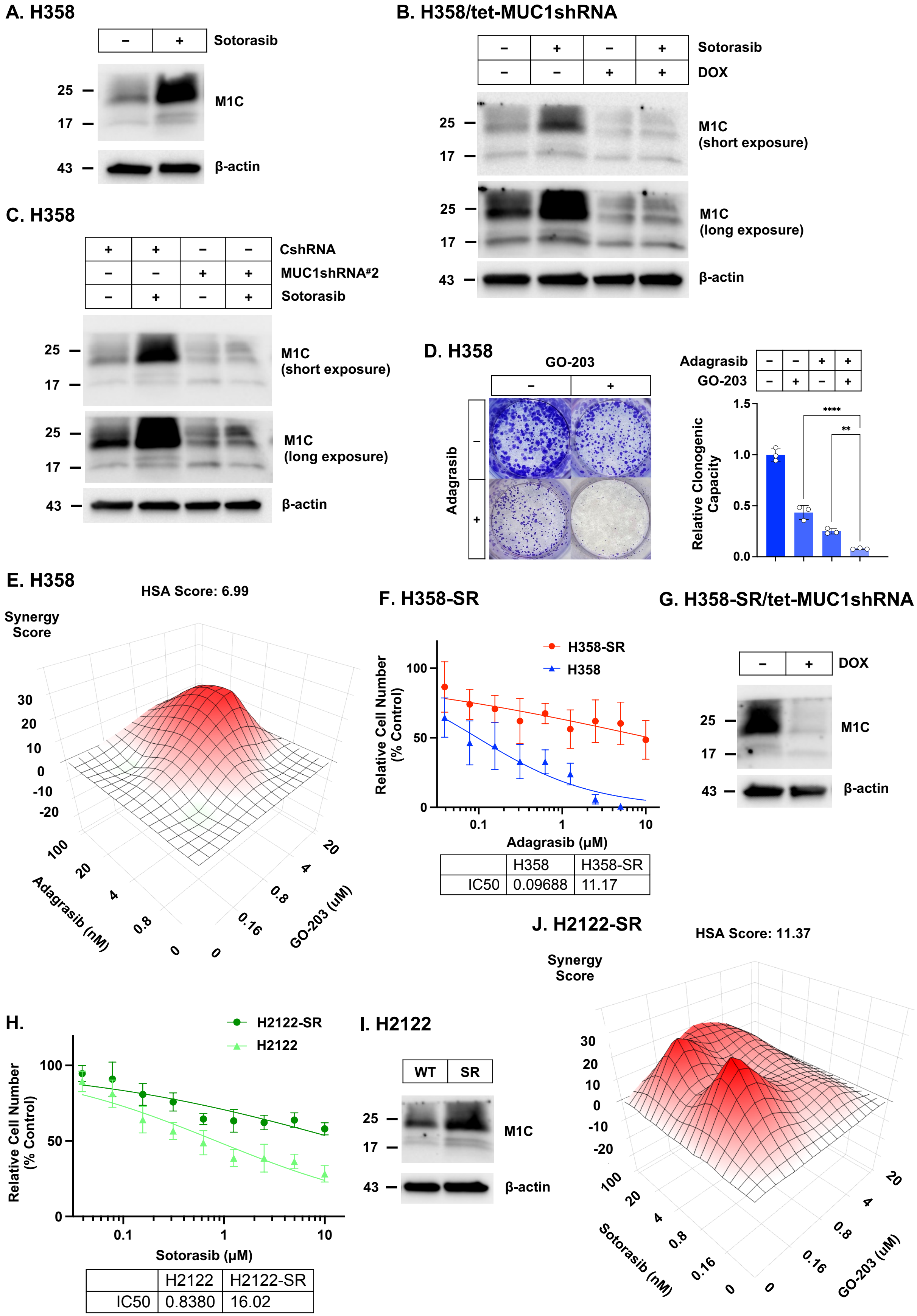


| | H358 | H358-SR |
|---|---|---|
| IC50 | 0.09688 | 11.17 |

| | H2122 | H2122-SR |
|---|---|---|
| IC50 | 0.8380 | 16.02 |
