# Supplementary material for: M1C is a druggable target for NSCLC KRAS G12C mutant tumors resistant to KRAS inhibitors
Source: Oncogene. 2026 Aug 5;45(38):4088–100. doi: 10.1038/s41388-026-03934-2 (PMC13577901; doi:10.1038/s41388-026-03934-2)
Supplement: Supplementary file 3 [file 41388_2026_3934_MOESM3_ESM.pdf]

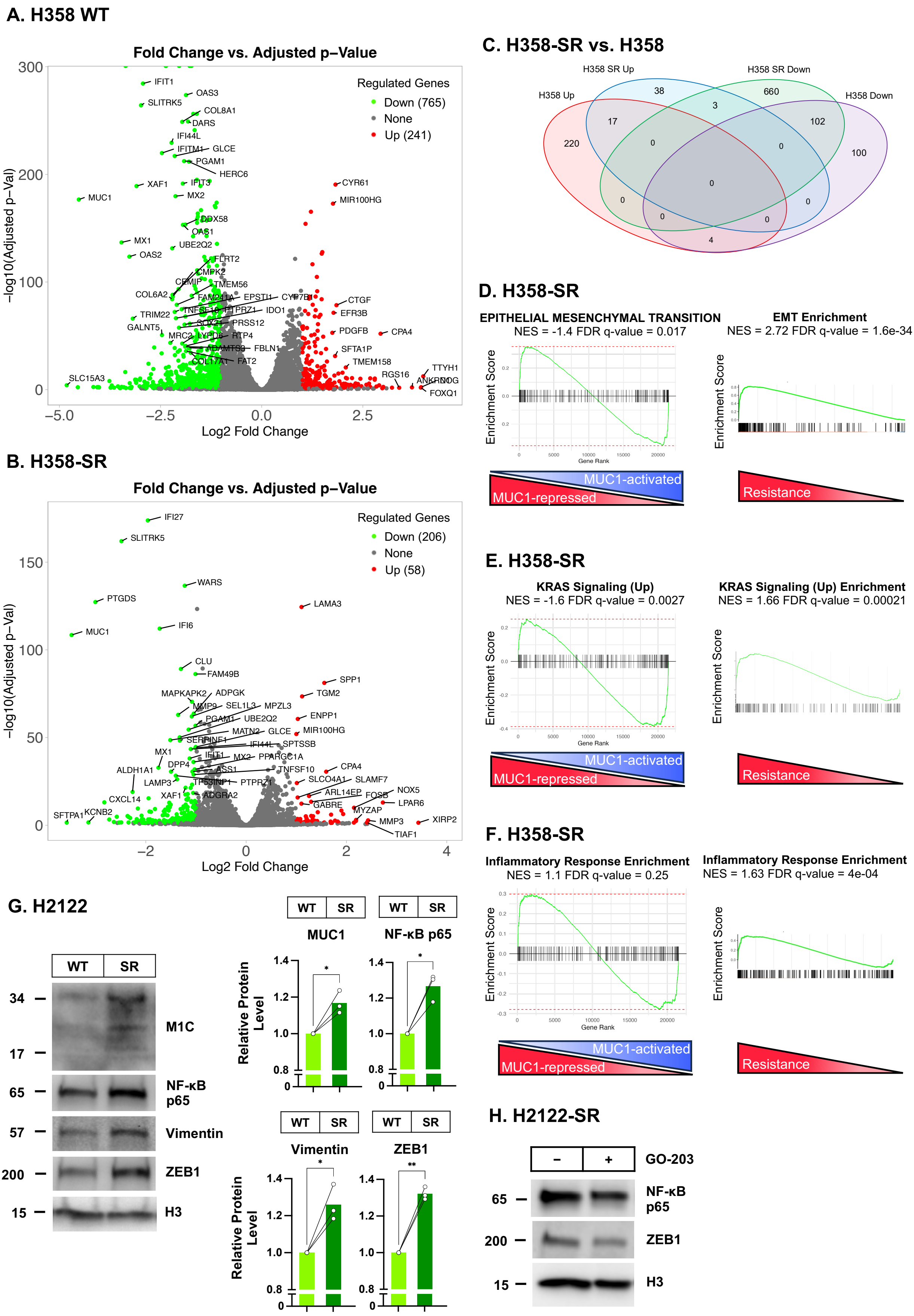
A. H358 WT
Fold Change vs. Adjusted p−Value
Regulated Genes
Down (765)
None
Up (241)
−log10(Adjusted p−Val)
Log2 Fold Change
B. H358-SR
Fold Change vs. Adjusted p−Value
Regulated Genes
Down (206)
None
Up (58)
−log10(Adjusted p−Val)
Log2 Fold Change
C. H358-SR vs. H358
H358 SR Up
H358 SR Down
H358 Up
H358 Down
D. H358-SR
EPITHELIAL MESENCHYMAL TRANSITION
NES = -1.4 FDR q-value = 0.017
EMT Enrichment
NES = 2.72 FDR q-value = 1.6e-34
Enrichment Score
Gene Rank
MUC1-activated
MUC1-repressed
Resistance
E. H358-SR
KRAS Signaling (Up)
NES = -1.6 FDR q-value = 0.0027
KRAS Signaling (Up) Enrichment
NES = 1.66 FDR q-value = 0.00021
F. H358-SR
Inflammatory Response Enrichment
NES = 1.1 FDR q-value = 0.25
Inflammatory Response Enrichment
NES = 1.63 FDR q-value = 4e-04
G. H2122
WT
SR
M1C
NF-κB p65
Vimentin
ZEB1
H3
MUC1
Relative Protein Level
H. H2122-SR
GO-203
