# Supplementary material for: M1C is a druggable target for NSCLC KRAS G12C mutant tumors resistant to KRAS inhibitors
Source: Oncogene. 2026 Aug 5;45(38):4088–100. doi: 10.1038/s41388-026-03934-2 (PMC13577901; doi:10.1038/s41388-026-03934-2)
Supplement: Supplementary file 4 [file 41388_2026_3934_MOESM4_ESM.pdf]

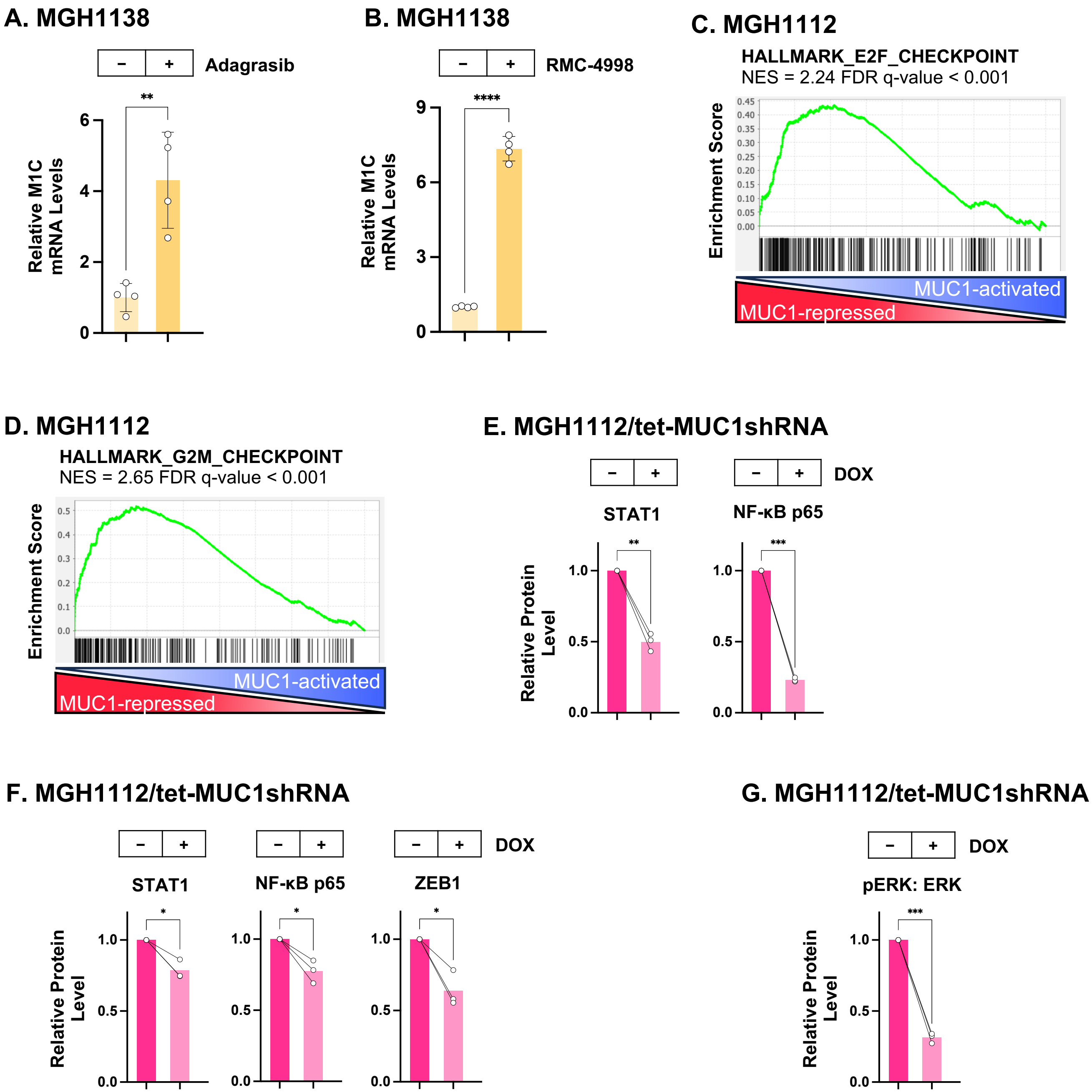
A. MGH1138
– + Adagrasib
**
Relative M1C mRNA Levels
0 2 4 6
B. MGH1138
– + RMC-4998
****
Relative M1C mRNA Levels
0 3 6 9
C. MGH1112
HALLMARK_E2F_CHECKPOINT
NES = 2.24 FDR q-value < 0.001
Enrichment Score
0.00 0.05 0.10 0.15 0.20 0.25 0.30 0.35 0.40 0.45
MUC1-activated
MUC1-repressed
D. MGH1112
HALLMARK_G2M_CHECKPOINT
NES = 2.65 FDR q-value < 0.001
Enrichment Score
0.0 0.1 0.2 0.3 0.4 0.5
MUC1-activated
MUC1-repressed
E. MGH1112/tet-MUC1shRNA
– + – + DOX
STAT1
NF-κB p65
**
***
Relative Protein Level
0.0 0.5 1.0
F. MGH1112/tet-MUC1shRNA
– + – + – + DOX
STAT1
NF-κB p65
ZEB1
*
Relative Protein Level
0.0 0.5 1.0
G. MGH1112/tet-MUC1shRNA
– + DOX
pERK: ERK
***
Relative Protein Level
0.0 0.5 1.0
