# Supplementary material for: M1C is a druggable target for NSCLC KRAS G12C mutant tumors resistant to KRAS inhibitors
Source: Oncogene. 2026 Aug 5;45(38):4088–100. doi: 10.1038/s41388-026-03934-2 (PMC13577901; doi:10.1038/s41388-026-03934-2)
Supplement: Supplementary file 5 [file 41388_2026_3934_MOESM5_ESM.pdf]

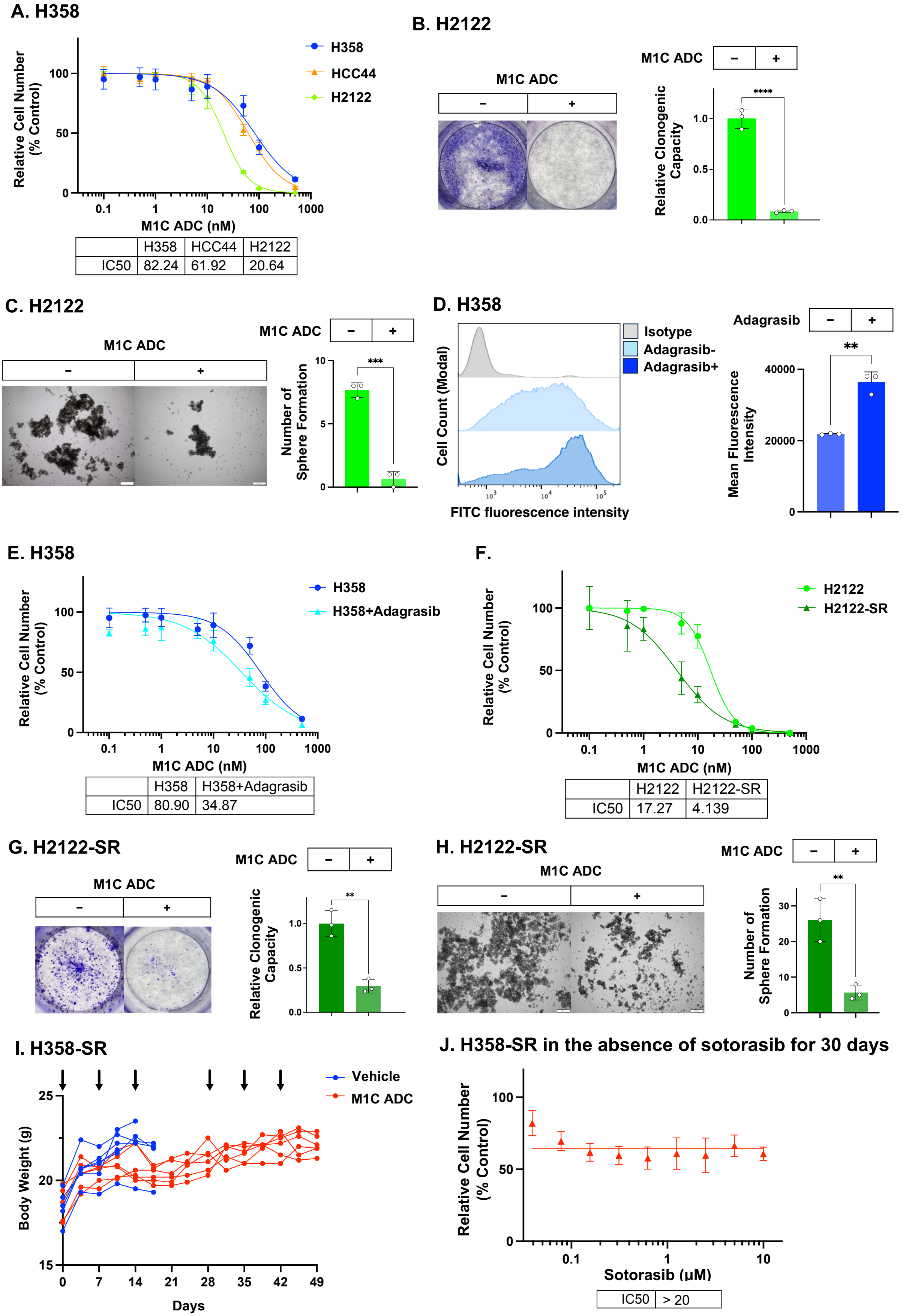
A. H358
H358
HCC44
H2122
Relative Cell Number (% Control)
M1C ADC (nM)
IC50 H358 82.24 HCC44 61.92 H2122 20.64
B. H2122
M1C ADC
–
+
Relative Clonogenic Capacity
****
C. H2122
M1C ADC
–
+
Number of Sphere Formation
***
D. H358
Isotype
Adagrasib-
Adagrasib+
Cell Count (Modal)
FITC fluorescence intensity
Adagrasib
–
+
Mean Fluorescence Intensity
**
E. H358
H358
H358+Adagrasib
Relative Cell Number (% Control)
M1C ADC (nM)
IC50 H358 80.90 H358+Adagrasib 34.87
F.
H2122
H2122-SR
Relative Cell Number (% Control)
M1C ADC (nM)
IC50 H2122 17.27 H2122-SR 4.139
G. H2122-SR
M1C ADC
–
+
Relative Clonogenic Capacity
**
H. H2122-SR
M1C ADC
–
+
Number of Sphere Formation
**
I. H358-SR
Vehicle
M1C ADC
Body Weight (g)
Days
J. H358-SR in the absence of sotorasib for 30 days
Relative Cell Number (% Control)
Sotorasib (μM)
IC50 > 20
