# Supplementary material for: M1C is a druggable target for NSCLC KRAS G12C mutant tumors resistant to KRAS inhibitors
Source: Oncogene. 2026 Aug 5;45(38):4088–100. doi: 10.1038/s41388-026-03934-2 (PMC13577901; doi:10.1038/s41388-026-03934-2)

A. MGH1112

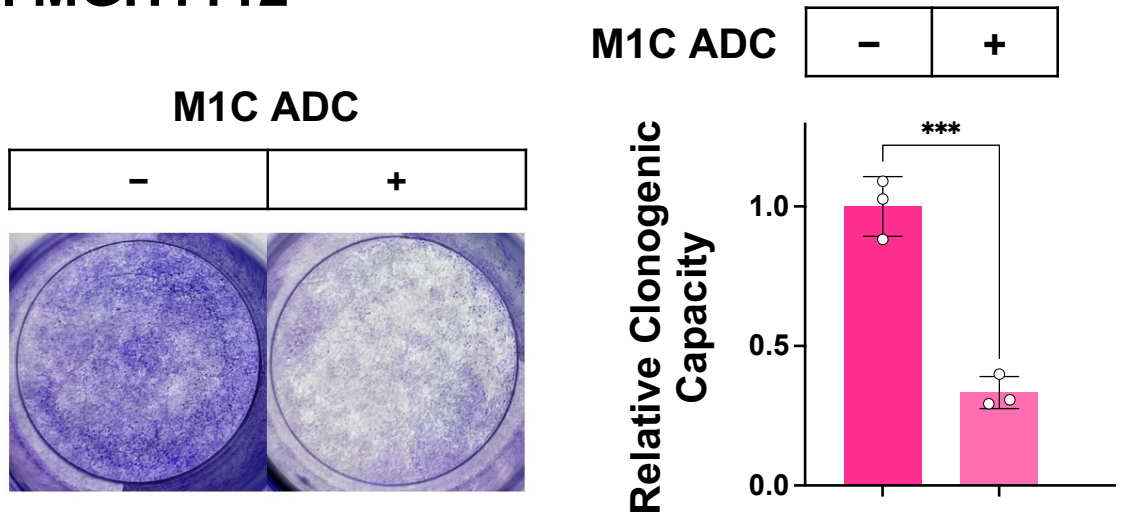

B. MGH1112

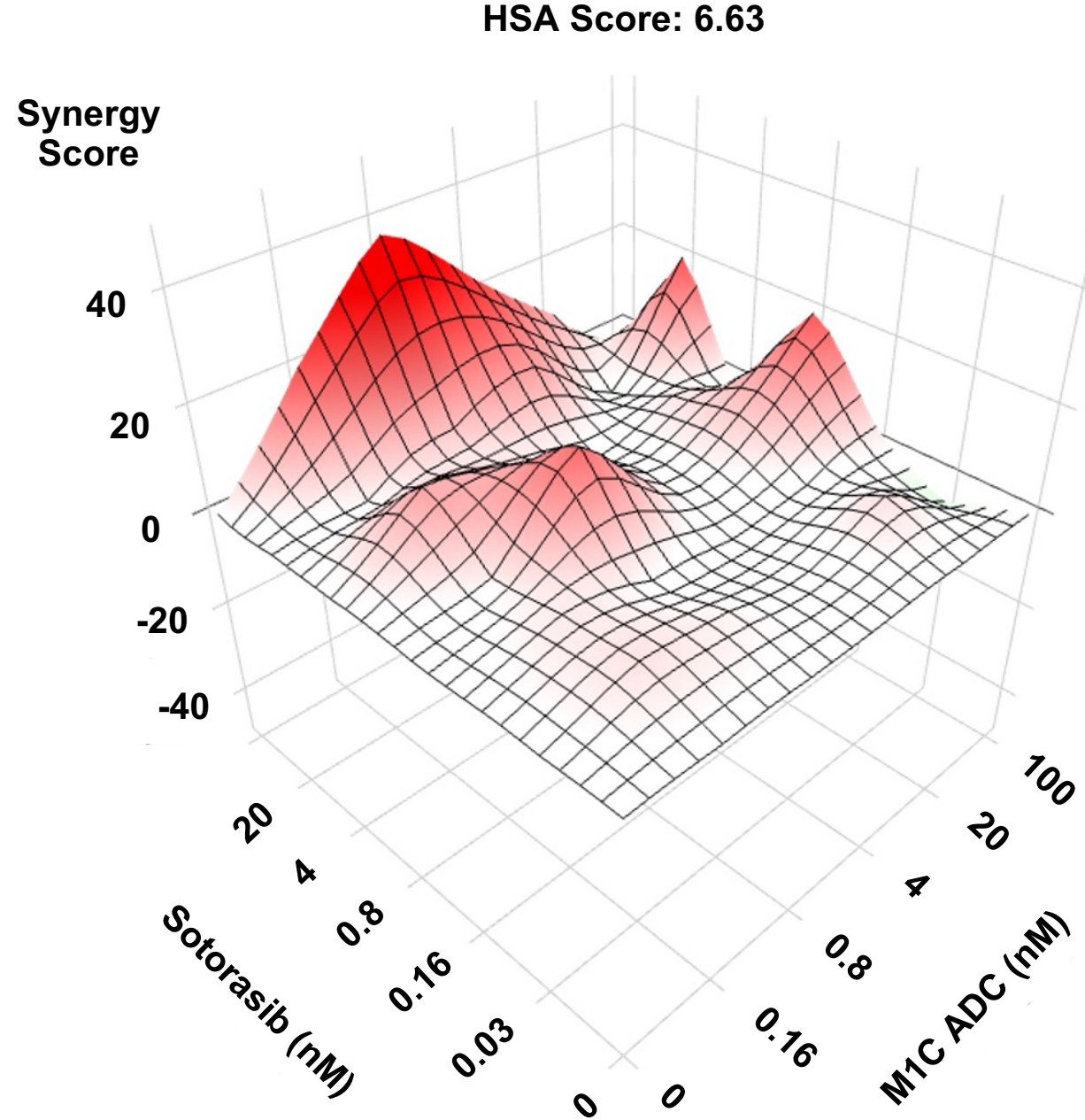

C. MGH1112

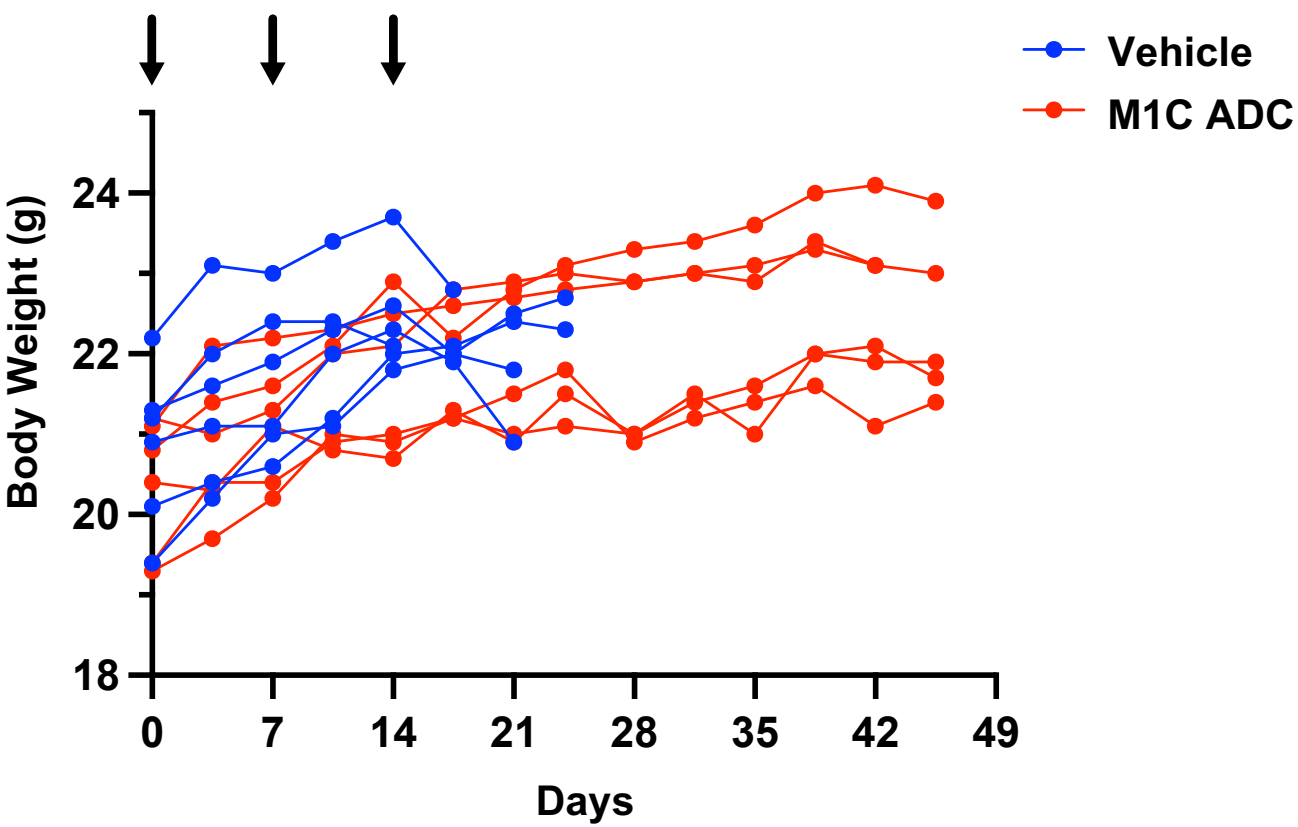

Supplement: Supplementary file 6 [file 41388_2026_3934_MOESM6_ESM.pdf]
