# Supplementary material for: M1C is a druggable target for NSCLC KRAS G12C mutant tumors resistant to KRAS inhibitors
Source: Oncogene. 2026 Aug 5;45(38):4088–100. doi: 10.1038/s41388-026-03934-2 (PMC13577901; doi:10.1038/s41388-026-03934-2)
Supplement: Supplementary file 7 [file 41388_2026_3934_MOESM7_ESM.docx]

**Supplemental Figure Legends**

**Supplemental Figure S1. Targeting KRAS induces M1C expression. A and B.** HCC44 (**A**) and H2122 (**B**) cells treated with 50 nM adagrasib and 50 nM MRTX1133 for 48 hours were analyzed for M1C transcripts. The results (mean±SD of 4 determinations) are expressed as relative levels compared to that obtained for control cells (assigned a value of 1). **C.** H1975 EGFR mutant cells treated with 50 nM osimertinib and 50 nM sotorasib for 48 hours were analyzed for M1C transcripts. The results (mean±SD of 4 determinations) are expressed as relative levels compared to that obtained for control cells (assigned a value of 1). **D.** H358 cells treated with 100 nM RMC-4998 for the indicated times were analyzed for M1C transcripts. The results (mean±SD of 4 determinations) are expressed as relative levels compared to that obtained for control cells (assigned a value of 1). **E.** Lysates from H358 cells treated with 50 nM sotorasib for 72 hours were immunoblotted with antibodies against the indicated proteins. **F.** H358 cells treated with 50 nM adagrasib for the indicated times were analyzed for STAT1 transcripts. The results (mean±SD of 4 determinations) are expressed as relative levels compared to that obtained for control cells (assigned a value of 1). **G.** Lysates from H358 cells treated with 50 nM adagrasib for 72 hours were immunoblotted with antibodies against the indicated proteins. **H.** Lysates from H358/CshRNA and H358/STAT1shRNA cells were immunoblotted with antibodies against the indicated proteins.

**Supplemental Figure S2. Targeting M1C reverses sotorasib resistance. A.** Lysates from H358 cells treated with 50 nM sotorasib for 48 hours and 100 nM RMC-4998 for 48 hours were immunoblotted with antibodies against the indicated proteins. **B.** Lysates from H358/tet-MUC1shRNA cells treated with vehicle or DOX for 7 days and then 50 nM sotorasib for 48 hours were immunoblotted with antibodies against the indicated proteins. **C.** Lysates from H358/CshRNA and H358/MUC1shRNA#2 cells treated with vehicle or 50 nM sotorasib for 48 hours were immunoblotted with antibodies against the indicated proteins. **D.** H358 cells treated with 0.5 μM GO-203 and 50 nM adagrasib for 7-14 days were analyzed for colony formation. The results (mean±SD of three determinations) are expressed as relative clonogenic capacity compared to that for untreated cells (assigned a value of 1). **E.** H358 cells were treated with the indicated concentrations of GO-203 and adagrasib for 3 days. Cell viability was assessed by Alamar Blue staining (mean of three determinations). Indicated are the combination indices as determined using HSA scores. **F.** H358 and H358-SR cells treated with the indicated concentrations of adagrasib for 3 days were assessed for cell viability by Alamar Blue staining (mean of six determinations). Indicated are the IC50 values. **G.** Lysates from H358-SR/tet-MUC1shRNA cells treated with vehicle or DOX for 7 days were immunoblotted with antibodies against the indicated proteins. **H.** H2122 and H2122-SR cells treated with the indicated concentrations of sotorasib for 3 days were assessed for cell viability by Alamar Blue staining (mean of six determinations). Indicated are the IC50 values. **I.** Lysates from H2122 and H2122-SR cells were immunoblotted with antibodies against the indicated proteins. **J.** H2122-SR cells were treated with the indicated concentrations of GO-203 and sotorasib for 3 days. Cell viability was assessed by Alamar Blue staining (mean of three determinations). Indicated are the combination indices determined using HSA scores.

**Supplemental Figure S3. M1C regulates transcriptomes of H358 and H358-SR cells. A and B.** Volcano plots of down- and upregulated genes in H358 (**A**) and H358-SR (**B**) cells with M1C silencing. **C.** Venn diagram of down- and up-regulated genes in H358 and H358-SR cells with M1C silencing. **D-F.** GSEA of H358-SR cells with M1C silencing (left) and comparison of H358-SR vs H358 enrichment using the indicated HALLMARK gene signatures. **G.** Chromatin from H2122 and H2122-SR cells was immunoblotted with antibodies against the indicated proteins (left). Relative protein levels were quantified by densitometry of three independent immunoblots (right). **H.** Lysates from H2122-SR cells treated with 5 μM GO-203 for 48 hours were immunoblotted with antibodies against the indicated proteins.

**Supplemental Figure S4. Activation of M1C in patient-derived MGH1138 and MGH1112 cells. A and B.** MGH1138 cells treated with 50 nM adagrasib (**A**) and 50 nM RMC-4998 (**B**) for 48 hours were analyzed for M1C transcripts. The results (mean±SD of 4 determinations) are expressed as relative levels compared to that obtained for control cells (assigned a value of 1). **C and D.** GSEA of RNA-seq data from MGH1112 cells with M1C silencing using the indicated HALLMARK gene signatures. **E and F.** Relative protein levels of STAT1 and NF-κB p65 in lysates (**E**) and STAT1, NF-κB p65, and ZEB1 in chromatin (**F**) from MGH1112/tet-MUC1shRNA cells treated with vehicle or DOX for 7 days. **G.** Relative pERK/ERK ratio in MGH1112/tet-MUC1shRNA cells treated with vehicle or DOX for 7 days.

**Supplemental Figure S5. M1C ADC is effective against sotorasib-resistant cells. A.** H358, HCC44 and H2122 cells treated with indicated concentrations of M1C ADC for 7 days were analyzed for cell viability by Alamar Blue staining. The results (mean±SD of six determinations) are expressed as relative cell number (% control) compared to that for untreated cells. Indicated are the M1C ADC IC50 values. **B.** H2122 cells treated with 30 nM M1C ADC for 7-14 days were analyzed for colony formation. Shown are representative photomicrographs of stained colonies (left). The results (mean±SD of three determinations) are expressed as relative clonogenic capacity compared to that for untreated cells (assigned a value of 1) (right). **C.** H2122 cells treated with vehicle or 50 nM M1C ADC for 7 days were analyzed for tumorsphere formation. Shown are representative photomicrographs of tumorspheres (left). The results (mean±SD of three determinations) are expressed as number of tumorsphere formations compared to that for vehicle treated cells (right). **D.** H358 cells left untreated or treated with 50 nM adagrasib for 2 days were analyzed by flow cytometry with a control IgG and anti-M1C. Shown are histograms of cells positive for M1C expression (left). The bar plot shows the mean fluorescence intensity (MFI), presented as the mean ± SD of three independent determinations (right). **E.** H358 cells treated with 50 nM adagrasib for 2 days and then M1C ADC for 7 days were analyzed for cell viability by Alamar Blue staining. The results (mean±SD of six determinations) are expressed as relative cell number (% control) compared to that for untreated cells. Indicated are the M1C ADC IC50 values. **F.** H2122 and H2122-SR cells treated with the indicated concentrations of M1C ADC for 7 days were analyzed for cell viability by Alamar Blue staining. The results (mean±SD of six determinations) are expressed as relative cell number (% control) compared to that for untreated cells. Indicated are the M1C ADC IC50 values. **G.** H2122-SR cells treated with 50 nM M1C ADC for 7 days were analyzed for colony formation. Shown are representative photomicrographs of stained colonies (left). The results (mean±SD of three determinations) are expressed as relative clonogenic capacity compared to that for untreated cells (assigned a value of 1) (right). **H.** H2122-SR cells treated with vehicle or 50 nM M1C ADC for 7 days were analyzed for tumorsphere formation. Shown are representative photomicrographs of tumorspheres (left). The results (mean±SD of three determinations) are expressed as number of tumorsphere formations compared to that for vehicle treated cells (right). **I.** Mean body weight changes of H358-SR tumor bearing mice treated with vehicle or M1C ADC. The results are expressed as the mean ratio of relative body weight for which the SEMs were <10%. **J.** H358-SR cells grown in the absence of sotorasib for 30 days were treated with indicated concentrations of sotorasib for 3 days and analyzed for cell viability by Alamar Blue staining. The results (mean±SD of six determinations) are expressed as relative cell number (% control) compared to that for untreated cells.

**Supplemental Figure S6. Effectiveness of the M1C ADC against MGH1112 cells and correlations of mucin gene expression in previously treated NSCLC KRAS G12C tumors. A.** MGH1112 cells treated with 100 nM M1C ADC for 7 days were analyzed for colony formation. Shown are representative photomicrographs of stained colonies (left). The results (mean±SD of three determinations) are expressed as relative clonogenic capacity compared to that for untreated cells (assigned a value of 1) (right). **B.** MGH1112 cells were treated with the indicated concentrations of M1C ADC and sotorasib for 7 days. Cell viability was assessed by Alamar Blue staining (mean of three determinations). Indicated are the combination indices determined using HSA scores. **C.** Mean body weight changes of MGH1112 tumor bearing mice treated with vehicle or M1C ADC. The results are expressed as the mean ratio of relative body weight for which the SEMs were <10%.
