# Supplementary material for: M1C is a druggable target for NSCLC KRAS G12C mutant tumors resistant to KRAS inhibitors
Source: Oncogene. 2026 Aug 5;45(38):4088–100. doi: 10.1038/s41388-026-03934-2 (PMC13577901; doi:10.1038/s41388-026-03934-2)
Supplement: Supplementary file 8 [file 41388_2026_3934_MOESM8_ESM.docx]

**Supplemental Table S1. Primers used for qRT-PCR analysis.**

| **M1C** | **FWD** | TACCGATCGTAGCCCCTATG |
| --- | --- | --- |
|  | **REV** | CTCACCAGCCCAAACAGG |
| **STAT1** | **FWD** | GGAACTTGATGGCCCTAAAGGA |
|  | **REV** | ACAGAGCCCACTATCCGAGACA |
| **NF-κB p65** | **FWD** | TAGGCGAGTTATAGCCTCAG |
|  | **REV** | CTGCAGTTTGATGATGAAGA |
| **ZEB1** | **FWD** | TCGGAAAGAGCTGTTCGCTT |
|  | **REV** | AGGAGGGGGCTGACATACAT |
| **MUC3** | **FWD** | AACTGGTGGAGAGCGTAGAG |
|  | **REV** | CGGTCCTGTAGCTTCTCACT |
| **MUC4** | **FWD** | AACACAGCCTGCTAGTCCAGCA |
|  | **REV** | TGGAGAGGATGGCTTGGTAGGT |
| **MUC13** | **FWD** | TCCAGCTGTTCAAGGTGCTA |
|  | **REV** | AGGCAGTGTTGGTAGAGGGA |
| **MUC16** | **FWD** | GCAGTGTGCTTGTGGACTCT |
|  | **REV** | GCCACAGTGATGTTGTAGCC |
| **MUC20** | **FWD** | CAAGATCACAACCTCAGCGA |
|  | **REV** | ACCTCCATTTTCACCTGCAC |
| **TFF1** | **FWD** | GGTCCTGGTGTCCATGCTG |
|  | **REV** | CACGAACGGTGTCGTCGAA |
| **β-actin** | **FWD** | GATGAGATTGGCATGGCTTT |
|  | **REV** | CACCTTCACCGTTCCAGTTT |
